# γδ T cells: origin and fate, subsets, diseases and immunotherapy

**DOI:** 10.1038/s41392-023-01653-8

**Published:** 2023-11-22

**Authors:** Yi Hu, Qinglin Hu, Yongsheng Li, Ligong Lu, Zheng Xiang, Zhinan Yin, Dieter Kabelitz, Yangzhe Wu

**Affiliations:** 1 Microbiology and Immunology Department, School of Medicine, Faculty of Medical Science, Jinan University, Guangzhou Guangdong, 510632 China; 2 Guangdong Provincial Key Laboratory of Tumour Interventional Diagnosis and Treatment, Zhuhai Institute of Translational Medicine, Zhuhai People’s Hospital Affiliated with Jinan University, Jinan University, Zhuhai Guangdong, 519000 China; 3 Department of Medical Oncology, Chongqing University Cancer Hospital, Chongqing, 400030 China; 4 Biomedical Translational Research Institute, Jinan University, Guangzhou Guangdong, 510632 China; 5 Institute of Immunology, Christian-Albrechts-University Kiel, Kiel, Germany

**Keywords:** Lymphocytes, Innate immunity, Adaptive immunity, Tumour immunology, Immunological disorders

## Abstract

The intricacy of diseases, shaped by intrinsic processes like immune system exhaustion and hyperactivation, highlights the potential of immune renormalization as a promising strategy in disease treatment. In recent years, our primary focus has centered on γδ T cell-based immunotherapy, particularly pioneering the use of allogeneic Vδ2^+^ γδ T cells for treating late-stage solid tumors and tuberculosis patients. However, we recognize untapped potential and optimization opportunities to fully harness γδ T cell effector functions in immunotherapy. This review aims to thoroughly examine γδ T cell immunology and its role in diseases. Initially, we elucidate functional differences between γδ T cells and their αβ T cell counterparts. We also provide an overview of major milestones in γδ T cell research since their discovery in 1984. Furthermore, we delve into the intricate biological processes governing their origin, development, fate decisions, and T cell receptor (TCR) rearrangement within the thymus. By examining the mechanisms underlying the anti-tumor functions of distinct γδ T cell subtypes based on γδTCR structure or cytokine release, we emphasize the importance of accurate subtyping in understanding γδ T cell function. We also explore the microenvironment-dependent functions of γδ T cell subsets, particularly in infectious diseases, autoimmune conditions, hematological malignancies, and solid tumors. Finally, we propose future strategies for utilizing allogeneic γδ T cells in tumor immunotherapy. Through this comprehensive review, we aim to provide readers with a holistic understanding of the molecular fundamentals and translational research frontiers of γδ T cells, ultimately contributing to further advancements in harnessing the therapeutic potential of γδ T cells.

## Introduction

Until now, cancer remains one of the biggest challenges for human health.^1^ The underlying cause is that cancer cells originate from healthy cells, which results in highly similar molecular fingerprints. This similarity makes it difficult for the immune system to recognize and efficiently kill transformed cells in a timely manner. Simultaneously, the unique microenvironment created by the transformed cells progressively attenuates immune functions, leading ultimately to immune escape.^2–4^ The imbalanced or exhausted immune system is widely acknowledged as one of the key physiological hallmarks of tumor patients, including reduced numbers of total leukocytes, dysfunctional γδ T cell subsets, and increased proportions of exhausted CD8^+^ T cells and Tregs, among others.^5–10^ Since 2016, we have been at the forefront of translating the application of allogeneic γδ T cells, specifically the Vγ9Vδ2^+^ γδ T subset, from the laboratory to clinical practice, with the aim of renormalizing the dysfunctional immune system in patients with advanced solid tumor^11,12^ or multidrug-resistant tuberculosis (MDR-TB).^13^ In a comprehensive study involving 132 patients diagnosed with various types of cancer (including liver, lung, pancreatic, breast, and others), we administered a total of 414 cell infusions. Through this investigation, we not only established the safety of transferring allogeneic Vγ9Vδ2^+^ γδ T cells (abbreviated as Vδ2 T cells below) generated from healthy donors’ PBMCs after in vitro expansion but also demonstrated their clinical efficacy in extending patient survival and improving quality of life.^11^ Nevertheless, while conducting this investigator-initiated clinical trial, it became apparent that patients’ responses to allogeneic Vδ2 T cell therapy varied, with some demonstrating favorable outcomes, while others experienced only modest improvement. This highlights the need to uncover the underlying factors that contribute to the failure of infused cells in inducing an immune response against cancer cells, particularly the adverse effects of the tumor microenvironment. In this comprehensive review, we explore the origin and fate of γδ T cells, their subsets, their relevance to various diseases including infections, autoimmune diseases, and cancer, as well as their functional differences, vulnerability, and transition within these contexts. Additionally, based on our insights and updated knowledge, we discuss and propose viable strategies for the application of allogeneic γδ T cells as promising immunotherapy for various diseases.

### γδ T cells vs αβ T cells

In the realm of cellular immunity, γδ T cells stand apart from their αβ T cell counterparts due to their distinct attributes, which intricately shape their roles in both pathogenesis of diseases and the field of immunotherapy. αβ T cells, forming the predominant subset of CD3^+^ T cells within the immune repertoire, predominantly recognize peptide antigens presented by major histocompatibility complex (MHC) molecules. In contrast, γδ T cells adopt an alternative T cell receptor (TCR) architecture, consisting of γ and δ chains, granting them the ability to perceive a wider array of antigens in MHC-independent manner. This remarkable property encompasses recognition of both exogenous and endogenous antigens, spanning foreign as well as self-antigens.^8,9,14^

This dichotomy between γδ and αβ T cells extends across numerous dimensions, encompassing TCR structural variances, thymic developmental trajectories, mechanisms of antigen identification and presentation, activation cues, their roles in pathological conditions, and their applications in immunotherapy. The structural divergence of the γδ TCR and αβ TCR serves as the bedrock for distinguishing the functional roles these two T cell subsets assume within immune responses. This distinction becomes particularly evident when delving into their respective thymic development, as elaborated upon in forthcoming sections. Specifically, while the developmental journey of αβ T cells entails stages of double-negative (DN), double-positive (DP), and single-positive (SP) prior to dissemination into the circulation, γδ T cells follow a distinct course. The latter either exit the thymus during the DN (DN2-DN3) phase or progress through DN and DP or DN, DP, and SP stages before embarking into circulation. This unique developmental pattern equips γδ T cells with a greater spectrum of immune functions, spanning both innate and adaptive roles, along with diversified capacities in antigen recognition and presentation.

Distinctly stratified in their antigen recognition, αβ T cells operate within the confines of MHC-dependent recognition, whereas γδ T cells extend their sensing capabilities to include stress-induced antigens, phosphoantigens, and other non-peptidic molecules, all while circumventing the need for MHC mediation. Yet, the most pivotal divergence lies in their activation mechanisms and antigen presentation capabilities. While activation of αβ T cells necessitates a dual input of signals—antigen recognition and co-stimulation—to orchestrate immune responses, γδ T cells can be activated by a singular signal,^15^ such as a phosphoantigen. Notably, within the realm of antigen presentation, a specific subset of human γδ T cells, the Vδ2 T cells, takes on the role of professional antigen-presenting cells (APCs), a function beyond the purview of αβ T cells. This unique attribute situates γδ T cells as key regulators of immune functions within the broader immune cell landscape.

In terms of disease, αβ T cells are well-known for their adaptive immune responses and are critical in combating infections and mounting antigen-specific immune responses.^9,16–18^ They are highly specialized and undergo clonal expansion upon encountering specific antigens, however, the frequency of cells among αβ T cells which can recognize a given peptide antigen is extremely low. In contrast, γδ T cells exhibit characteristics of both innate and adaptive immunity. They can rapidly respond to various pathogens through their innate-like receptors, allowing for early immune defense even in the absence of prior antigen exposure.^9,14^ Moreover, γδ T cells are actively involved in tissue surveillance at barrier sites and contribute to the maintenance of tissue homeostasis.^10,19^ In disease settings, γδ T cells have been implicated in both protective and pathogenic roles. Their ability to respond rapidly to infections and produce cytokines enables them to contribute to pathogen clearance. However, dysregulation of γδ T cell activation and function has been associated with the development of autoimmune diseases, where these cells can recognize self-antigens and contribute to tissue damage.^7,20^

In the field of immunotherapy, αβ T cells have been extensively studied fundamentally and clinically, and the paradigm is chimeric antigen receptor (CAR)-T cell therapy, which targets specific antigens on cancerous or autoreactive immune cells.^21–24^ In comparison, γδ T cells are relatively less explored but show great potential. Due to their innate-like features, γδ T cells have the capacity to recognize and eliminate tumor cells without prior sensitization. This makes them attractive candidates for immunotherapeutic strategies, including administration of freshly expanded^11,12^ and genetically modified γδ T cells (e.g. CAR-γδ T).^25–27^

The multifaceted functions and extensive antigen recognition abilities of γδ T cells, coupled with their unique properties such as innate and adaptive-like traits and the capacity to identify stress-induced molecules, render them invaluable in the context of diseases and as prime candidates for immunotherapeutic strategies. To benefit further research on the mechanisms underlying γδ T cell roles in disease and on the optimization of its therapeutic potential, we comprehensively reviewed the origin and fate, γδ T cell subsets, and their roles in diseases and immunotherapy.

### Chronological milestones of γδ T cell research

**Fig. 1:**
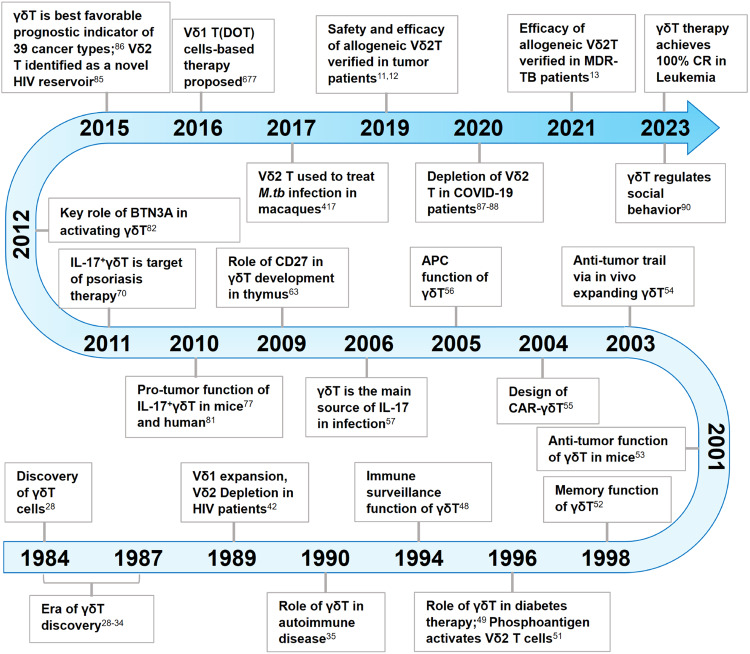
Chronological Milestones of γδ T cell research from its discovery in 1984 till 2023. HIV human immunodeficiency virus, CAR chimeric antigen receptor, APC antigen-presenting cell, IL-17 interleukin 17, BTN3A butyrophilin 3A, *M.tb* mycobacterium tuberculosis, MDR-TB multidrug-resistant tuberculosis, CR complete remission, COVID-19 coronavirus disease of 2019

To help readers better establish a whole picture about the discovery and the roles of γδ T cells in diseases, we summarize the milestones about γδ T cells since their discovery from the beginning of 1980s (Fig. 1). In fact, it came as a big surprise when the existence of a second set of rearranging TCR genes was discovered. In 1984, γδ T cells were first reported by Tonegawa et al.,^28^ with significant contributions from Adrian C. Hayday.^28,29^ Until 1987, important work on identifying γ and δ chains and their rearrangements marked a key era in γδ T cell discovery.^28–34^ These foundational discoveries have laid the groundwork for comprehending their distinctive attributes and have opened doors for more extensive investigations into their functional roles within the immune system and across diverse disease contexts. Starting in 1989, studies began to unravel the involvement of γδ T cells in autoimmune diseases and anti-infection immunity.^35–44^ Notably, in human immunodeficiency virus (HIV) infected patients, a shift in the γδ T cell subtypes was observed, with an expansion of Vδ1^+^ and depletion of Vδ2^+^ subtype, leading to an inversion of Vδ2/Vδ1 ratios in circulating γδ T cells.^42,45–47^ These findings provided important insights into the dysregulation of γδ T cell populations in specific diseases.

In the following years, the multifaceted functions of γδ T cells were further elucidated. In 1994, the role of intraepithelial γδ T cells in immune surveillance and tissue repair was reported,^48^ highlighting their significance in monitoring and maintaining the integrity of damaged epithelial tissues. Additionally, the therapeutic potential of γδ T cells was demonstrated in mouse models of diabetes and allergic airway inflammation.^49,50^ Furthermore, the discovery of the activation of Vδ2 T cells by so-called ‘phosphoantigens’ opened avenues for exploring the unique activation mechanisms of γδ T cells,^51^ and the memory function^52^ demonstrated the adaptive immunity of γδ T cells.

From 2001 to 2010, several milestones were achieved in the field of γδ T cell research. The anti-tumor function of γδ T cells was discovered in murine models, leading to their use as immunotherapy for lymphoid malignancies.^53,54^ Genetic modification techniques were applied to enhance the cytotoxicity of γδ T cells, pioneering the design of CAR-γδ T cells.^55^ Furthermore, γδ T cells were found to serve as professional APCs,^56^ expanding our understanding of their immune regulatory functions. During this period, IL-17-producing γδ T cells gained great attention,^57–67^ particularly in the context of infectious and inflammatory diseases. Their crucial roles in immune responses to pathogens, such as *Mycobacterium tuberculosis* (*M.tb*) and *Escherichia coli*, were elucidated.^57–60^ Moreover, the association between γδ T cells and autoimmune inflammation was established,^62,68^ further underscoring their diverse functions in immune homeostasis.

In the subsequent years, research efforts focused on IL-17-producing γδ T cells and their implications in human diseases. Their involvement in psoriasis, as both necessary and sufficient for plaque formation, identified IL-17-producing γδ T cells as promising therapeutic target.^69–75^ Notably, the pro-tumor role played by IL-17^+^γδ T cells in both mice^76–79^ and humans^80,81^ has significantly enhanced our understanding of the involvement of γδ T cells in tumorigenesis. A significant breakthrough was the discovery of BTN3A1^82^ as a sensing molecule for phosphoantigens which added substantially to our understanding of the mechanisms of activation of human γδ T cells. Furthermore, the identification of memory γδ T cells revealed their adaptive functions and immune memory capabilities.^83,84^ Additionally, γδ T cells’ adverse role as a host for HIV latent infection^85^ and their prognostic value in various types of cancer^86^ highlighted their broader clinical significance.

In recent years, groundbreaking advancements have been made in the clinical application of γδ T cells. Studies from our group demonstrated the safety and efficacy of allogeneic Vδ2 T cells derived from healthy donors in the treatment of late-stage lung and liver cancer patients,^11,12^ pointing towards the potential of off-the-shelf Vδ2 T cell products in cancer immunotherapy. Furthermore, the successful application of γδ T cells in treating MDR-TB^13^ and their functional attenuation in COVID-19 patients^87–89^ expanded our understanding of their therapeutic potential in combating challenging infectious diseases. Strikingly, the regulatory role of γδ T cells in the social behavior of mice^90^ and the achievement of 100% complete remission in leukemia patients (NCT03533816) who received γδ T cell therapy have heightened our anticipation regarding the physiological functions of γδ T cells.

In summary, the chronological progression of γδ T cell research has revealed their diverse functions and therapeutic implications in autoimmune diseases, infections, and cancers. From understanding their roles in immune surveillance and tissue repair to their applications in immunotherapy and disease management, γδ T cells have emerged as important players in the field of immunology. Continued research in this area holds great promise for the development of novel therapeutic strategies and improved patient outcomes.

## γδ T cell origin and development

### γδ T cell origin

Like αβ T cells, γδ T cells develop in the thymus from progenitor T cells originating from bone marrow hematopoietic stem cells. They are considered the earliest T cell subset in vertebrates. In murine models, γδ T cell development in the thymus has been extensively studied,^91–93^ revealing that DN (CD4^−^CD8^−^) cells expressing TCR-γδ commit to the γδ lineage without undergoing DP (CD4^+^CD8^+^) selection. Conversely, for αβ T cells, DN cells expressing the pre-TCR (TCR‐β paired with the invariant pre‐TCR‐α chain) develop through DP and then differentiate into SP (CD4^+^ or CD8^+^) cells.^16,92,94–98^ Evidence suggests that γδ T cell development in the human thymus follows a similar pattern,^93^ although the regulatory mechanisms, including signaling, factors, and molecular processes controlling V(D)J rearrangement, require further confirmation. Often, these mechanisms are investigated based on findings from murine studies.^95^ Nonetheless, more and more insights into the development of human γδ T cells in the thymus are gradually accumulating.

During thymic development, γδ T cells precede αβ T cells in ontogeny, and γδ TCR rearrangements occur early in embryonic stages in mice and humans.^99–101^ γδ versus αβ T cell commitment depends on TCR signal strength and Notch signaling.^102–104^ In mice, strong TCR signaling without Notch signal induces γδ lineage commitment, while low TCR signal strength with strong Notch signaling promotes αβ lineage.^105–107^ Notch signaling alone is insufficient to determine γδ/αβ commitment. Intrinsic signals from the TCR complex, along with trans-conditioning by different thymocyte subsets, also contribute to this process.^108^

**Fig. 2:**
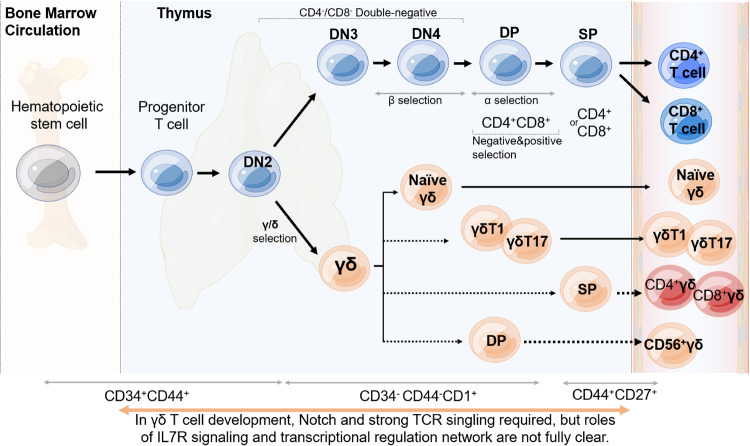
The possible mechanisms of human γδ T development and fate decision in thymus. In circulation, the phenotypes of γδ T cells at least include naïve γδ T cells, IFNγ-producing γδ T cells (γδT1), IL-17-producing γδ T cells (γδT17), IFNγ/IL4-producing γδ T cells (γδNKT or CD56^+^γδT), very rare CD4^+^γδ T cells, and CD8^+^γδ T cells

In humans, sustained Notch signaling is required for γδ T cell development, mediated by specific Notch receptor–ligand interactions, particularly Jagged2/Notch3 signaling.^109,110^ Human γδ T cell differentiation involves a Notch-independent DN pathway generating mature DN and SP (CD8^+^) γδ T cells, and a Notch-dependent DP pathway producing immature CD4^+^ SP cells followed by DP γδ T cells. The postnatal human thymus exhibits DN, DP, and SP TCRγδ^+^ populations, highlighting heterogeneity.^97,103,111^ Although only a small fraction of γδ T cells co-express either CD8 or CD4 (SP) on their surface, with CD8^+^γδ T cell population being the most abundant, this implies a fraction of γδ T cells undergo the similar DP to SP development route as αβ T cell since they share the same co-receptor CD8 and CD4. This observation is puzzling since unlike αβ T cells, γδ T cell mediated recognition is MHC non-restricted, therefore, the exact role of CD8 or CD4 expression on γδ T cells and precise ontogenesis of thymic γδ T cells awaits further elucidation. Notably, growing evidence has revealed that circulating γδ T cells also express high level of CD56, endorsing γδ T cells phenotypically similar to natural killer T-cells (NKT), which mature in thymus at the DP stage. Whether or not and how CD56^+^γδ T (γδNKT) cells^112,113^ mature at the DP stage remain mysterious and to be fundamentally resolved. Collectively, the above discussions are briefly sketched in Fig. 2. Additionally, activated extrathymic γδ T cells express Notch receptors, regulating effector functions. Inhibiting Notch signaling has been shown to impair the anti-tumor cytotoxicity of γδ T cells, providing further evidence of its significance in both thymic development and overall function.^114^ The human γδ T cell repertoire undergoes diversification at birth, with the Vδ1^+^ subset dominating in cord blood. However, as individuals mature into adulthood, this repertoire becomes more constrained, and the Vγ9Vδ2 subset takes precedence in peripheral blood, constituting 75% or more of the γδ T cell population.^99^ Additionally, the Vδ1^+^ subset was also found to be enriched in the post-natal thymus, demonstrating thymic rearrangement and expression of *TRG* and *TRD* genes.^115^ This finding supports previous conclusions regarding the TCR repertoire of γδ T cells that develop in the human thymus. Altogether, understanding γδ T cell development illuminates their roles in immune surveillance and responses, providing insights into regulatory mechanisms and heterogeneity within this T cell subset.

### γδ-TCR V(D)J recombination

Overall, γδTCR expression was detected by 14 days of gestation in murine^100^ and by eight weeks of fetal development in human.^116^ They constitute the initial T cell lineage to undergo development within the thymus and then migrate to various tissues, where they serve as swift producers of effector cytokines like IFNγ and IL-17, crucial for barrier defense. The divergence between γδ and αβ T cells takes place during their development in the thymus at the DN stage. At this stage, thymocytes evolve into two distinct T cell lineages based on the expression of either γδ or αβ TCRs.^117–120^ Most of the γδ T cells remain DN and develop into mature γδ T cells before they egress from the thymus.

The generation of a diverse TCR repertoire involves the V(D)J recombination of the four TCR loci. This recombination occurs at different stages of thymocyte development, with *TRB, TRG*, and *TRD* loci rearranging in the CD34^+^ stages, and *TRA* rearranging in the DP stage.^121,122^ Rearrangement of the *TRG* locus happens earlier and is potentially completed earlier than the *TRB* locus, indicating sequential and overlapping rearrangement windows. The human *TRG* locus consists of 14 *TRGV* genes (of which only six are functionally expressed; Vγ2-5, Vγ8, Vγ9) and 5 *TRGJ* genes (JP1, JP, J1, JP2, J2), which can associate with one of two *TRGC* elements.^9^ During fetal development, central *TRGV* elements are predominantly rearranged, while postnatal thymocytes mainly use distal *TRGV* and *TRGJ* segments with *TRGC2*.^123,124^ The *TRD* locus contains eight *TRDV* segments, of which *TRDV4-8* also have *TRA* designation due to their location within the *TRA* locus. The usage of V segments in V(D)J recombination changes during development, with fetal thymocytes favoring downstream *TRDV* and *TRDJ* segments and a shift towards more upstream elements occurring later in life.^95,124,125^ It should be marked here that one major distinction of γδ T cells from conventional αβ T cells, is the diversity of TCR sequences endowed by the recombination activating gene (*RAG*)-mediated V(D)J recombination of TCRδ (*TRD*) locus (*TRDV, TRDD, TRDJ*) and TCRγ (*TRG*) locus (*TRGV, TRGJ*), similar to the TCRβ locus (*TRB*) and TCRα (*TRA*) of αβ T cells.^9^ Despite the low number of functionally expressed Vγ and Vδ genes (see above), theoretically, γδ T cells can generate up to 10^17-10^18 γδTCRs due to non-germline encoded variability occurring during recombination,^14,126^ compared with αβ TCRs, which can generate 10^15-10^18 αβTCRs. However, in reality, most of the peripheral Vδ2 T cells display semi-invariant TCR repertoires, using the same Vγ9 gene segments in both cord and adult blood.^127,128^ This may be due to continuous microbial exposures after birth, leading to the focusing of Vγ9Vδ2 T cell repertoire among individuals.^14,128,129^ Moreover, the reduction of γδTCR diversity in cancer patients^130^ suggests that tumor antigen recognition can also result in clonal focusing of the γδ TCR repertoire.

In human, the incorporation of nucleotides during V(D)J recombination varies between embryonic, fetal, and postnatal γδ thymocytes. Fetal thymocytes, characterized by delayed induction of terminal deoxynucleotidyl transferase (TdT), exhibit highly invariant germline-encoded complementarity-determining region-3 (CDR3) sequences in γδ T cells generated during early development. The expression of TdT is regulated by the RNA-binding protein LIN28B, which is abundantly expressed in fetal γδ T cells and acts as an inhibitor of TdT. In the absence of TdT, short homology repeats present in certain V/D/J segments can facilitate recombination, resulting in the formation of specific germline-encoded sequences in fetal γδ thymocytes. This differential regulation of TdT and the utilization of short homology repeats are responsible for the generation of invariant/public cytomegalovirus (CMV)-reactive *CDR3* sequences and the acquisition of effector functions in the fetal γδ T cell repertoire. These distinct characteristics are attributed to the intrinsic properties of fetal hematopoietic stem and precursor cells, characterized by high expression of LIN28B, and are dependent on the HSPC/LIN28B axis within the human fetal thymus.^115,124,131,132^ Notably, γδ-TCR recombination involves strict regulation, the allelic exclusion, which refers to the process of achieving monoallelic expression of a gene. While biallelic rearrangements have been observed at the *TRD* locus, they are less frequent and mostly represent incomplete or out-of-frame rearrangements. In contrast, functional rearrangements at both *TRG* alleles suggest allelic inclusion for this locus, allowing the expression of two different γ-chains on the same cell.^133–135^

### γδ-TCR V(D)J recombination signaling

The factors and molecular processes governing V(D)J recombination at the *TRD* and *TRG* loci in humans are not fully understood, but studies in mice suggest IL7R signaling, E proteins (HEB and E2A), Notch signaling, and transcription factors MYB and RUNX1 play crucial or important roles in regulation of *TRD/TRG* rearrangement.^91,102–104,109,136,137^ For IL7R signaling, its role in regulating *Trg* rearrangement has been mainly documented in murine. In human, however, even though it has been implicated in the regulation of *TRG* rearrangement as well, further evidence is required. IL7R signaling induces histone acetylation, chromatin accessibility, transcription, and rearrangement at the *Trg* locus through IL7-induced recruitment of STAT5 to the *Trg* enhancer Eγ.^138–140^ E proteins (HEB and E2A) play a crucial role in regulating V(D)J recombination at the *TRG* and *TRD* loci. They can induce recombination at the human *TRG* and *TRD* loci in non-lymphoid cells, likely by controlling accessibility at recombination signal sequence (RSS) sites.^141–143^ Notch signaling, in addition to its positive effects on TCR rearrangement, can negatively control the process by inhibiting E protein function and promoting degradation of E2A, and can upregulate MYB and RUNX1, which are involved in promoting chromatin accessibility and germline transcription at the *TRG* and *TRD* loci.^144,145^ These pathways and transcription factors are interconnected, as shown by Notch-mediated induction of MYB and RUNX1, which in turn regulate the accessibility and transcriptional activity of the *TRG* and *TRD* loci. MYB and RUNX1 can promote chromatin accessibility by recruiting histone-modifying enzymes and chromatin remodeling complexes. Additionally, epigenetic modifications and lineage-specific factors may also play roles in regulating V(D)J recombination.^91,95,141^ Overall, the regulation of V(D)J recombination at the *TRG* and *TRD* loci involves a complex interplay of various signaling pathways, transcription factors, epigenetic modifications, and lineage-specific factors. Further research is still needed to fully understand the precise mechanisms underlying the regulation of TCR gene rearrangement in humans.

### γδ selection and fate decision

As one subset of T lymphocytes, γδ T cells also develop from hematopoietic stem and progenitor cells (HSPCs) found in the bone marrow or fetal liver. These HSPCs migrate to the thymus as multipotent thymus seeding progenitors (TSPs) and undergo a complex differentiation process under the influence of the thymic microenvironment. TSPs can also develop into other cell types such as natural killer (NK) cells and dendritic cells (DCs) under specific culture conditions.^95,98,110,121,146^ Notch signaling, triggered by interaction with Notch ligands on thymic epithelial cells (TECs), leads to the progression of TSPs to the early T cell precursors (ETPs) stage,^102,109–111,147^ accompanied by the upregulation of genes like *GATA3*^146^ and Interleukin 7 receptor (*IL7R*)^104,111,136^ crucial for T cell development. ETPs exhibit limited potential to develop into other cell lineages,^104,109,147,148^ and the transcription factors BCL11B and GATA3 further promote the T cell lineage while suppressing alternative cell fates.^146,149^ The upregulation of CD1 and recent identification of CD44 loss^150^ serve as markers of irreversible commitment to the T cell lineage. It is noteworthy to mention that CD44^dim^ expression is observed in normal uncommitted ETPs. The loss of CD44, manifested in terms of gene and protein levels, takes place during the double-negative (DN) stage prior to CD1a surface expression.^150^ Consequently, the downregulation of CD44 has been recognized as a pivotal and accurate indicator of T-cell commitment (Fig. 2).^95^ IL7 signaling, induced by TEC-derived IL7, supports the proliferation and survival of T lineage cells,^104,137^ as evident from IL7R-deficient patients^151^ lacking T cells. Once committed, T lineage cells can differentiate into either αβ or γδ lineage T cells but lose their potential to develop into non-T lineage cells. Determining the exact stage at which bi-potent progenitors commit to either αβ or γδ lineage has been challenging in human, and the precise definition of these lineages has been ambiguous as well. Thus the γδ T cell receptor has been used as a reliable marker for γδ fate, since no unique surface marker other than TCR has been identified for γδ T cells, and the limited enriched cell surface markers in particular developmental stages are different between murine and human.^93,95^ Additional complexity arises from the observation that human γδ lineage cells can differentiate through a transient DP stage.^103^ Lastly, human fetal γδ T cells exhibit a phosphoantigen-reactive TCR repertoire, but the role of endogenous phosphoantigens is uncertain. Ligand-independent TCR signaling, analogous to pre-TCR signaling, potentially influences γδ lineage commitment in humans. Even more complexity is added by the fact that trans-rearrangements between TCR loci have been identified, giving rise to rare αβ T cells which express a Vγ instead of a Vβ gene.^152,153^, Furthermore, allelic exclusion between TCR αβ vs. γδ genes is not complete, since small number of T cells simultaneously expressing functional αβ and γδ TCRs are present in healthy donors and patients with autoimmune diseases.^154^ Excitingly, application of advanced technology such as single-cell transcriptome and proteome will significantly benefit the establishment of clear lineage-specific gene expression signatures and the identification of unique surface markers, which will promisingly promote our understanding about γδ T cell fate determination in thymus.^93,155,156^ For instance, a recent research using single-cell RNA sequencing (scRNA-seq) and high-dimensional flow cytometry has provided an updated insight into the developmental trajectory of Vγ9Vδ2 T cells within the postnatal thymus.^157^ This trajectory has been delineated into three discrete stages, characterized by the acquisition of functionality and substantial alterations in the expression patterns of transcription factors, chemokines, and surface markers. Specifically, these stages are demarcated as follows: stage 1 cells, identifiable by CD4^+^CD161^−/low^ markers; stage 2 cells, characterized as CD4^−^CD161^−^; and stage 3 cells, distinguished by CD4^−^CD161^+^ markers. This work offered a foundational understanding for future investigations into influential factors shaping the development of human γδ T cells in thymus, and particularly enhanced our comprehension of the molecular mechanisms steering human Vγ9Vδ2 T cell development, which would potentially facilitate Vγ9Vδ2 T cell-based immunotherapy in the context of diseases like cancer and infections.

### γδ cell fate decision signaling

About the regulation signaling of γδ cell fate decision, serval molecular mechanisms are involved, mainly including TCR signaling, Notch signaling, IL7R signaling, and the transcriptional regulation network (Fig. 2). For the role of TCR signaling^158^ in deciding αβ fate, two models were proposed: instructive (strength of TCR signaling determines fate) and stochastic (random occurrence from DP to SP).^159^ For γδ fate decision, it appears to be predetermined rather than randomly occurred in mice based on DN thymocytes expressing high levels of SOX13 or IL7R.^160,161^ Studies indicate that lineage choice is also determined by the TCR signal strength rather than TCR type. γδ cells exhibit stronger TCR signaling compared to αβ cells, which influences gene expression and cell fate.^105,107,119,162^ This has been confirmed through manipulations of signal strength, where the γδ TCR activates stronger MAPK signaling, resulting in prolonged ERK activation and stabilization of EGR1.^105,163–165^ Differences in downstream components and the abundance of γδ TCR contribute to signal intensity. Similar mechanisms are suggested to operate in human thymocytes, where chromatin changes and AP-1 motifs are associated with γδ commitment.^166,167^ TCR signaling prevents the transition to the αβ lineage and instead induces γδ-like cells in thymus. Upregulation of EGR transcription factors and ID3 further support the role of signal strength as an instructive factor.^105,167^ While the instructive model likely applies to human γδ T cell development, further research is needed to confirm its validity.

Notch signaling and IL7R signaling play distinct roles in γδ T cell development, with species-specific requirements.^102–104,109–111,114,136,138,145,147,148^ In mice, Notch signaling promotes αβ lineage development, while in humans, evidence suggests its involvement in favoring the γδ lineage. Notch ligands, particularly JAG2, support γδ T cell development, while DLL1 and DLL4 contribute to αβ lineage development. The molecular mechanisms underlying the preference for γδ fate remain unclear, but Notch signaling counteracts the αβ lineage transcription factor BCL11B. On the other hand, IL7 signaling exhibits species-specific effects. In mice, deficiencies in the IL7 pathway significantly impair γδ lineage development, while the impact on αβ lineage is moderate. In humans, even though several studies indicated that inhibiting IL7R disrupts αβ lineage development but allows reduced γδ differentiation,^95,104,137^ the in-depth role of IL7R signaling in human γδ lineage commitment requires further investigation.

Identifying the transcription factors involved in establishing γδ fate has been a challenging task as well. Although a transcriptional signature of mouse γδ thymocytes has been described, many factors were also found in other T cell types.^95^ EGR1-3 and ID3 are potential regulators induced by TCR signaling, with ID3 inhibiting T lineage commitment and *TRD* rearrangements.^105,107,163,168^ SOX13 is involved in γδT17 differentiation, while RUNX3’s specific functions in γδ lineage commitment remain unclear. Other factors, such as NR4A1-3, ETV5, KLF2, RELB, HES1, and ZBTB16, are selectively upregulated in human γδ lineage thymocytes.^167,169,170^ Epigenetic regulation varies between αβ and γδ committed cells, with γδ T cells exhibiting extensive chromatin remodeling.

In conclusion, γδ cell fate regulation involves intricate interplay among TCR, Notch, and IL7R signaling pathways, along with a complex transcriptional network. While TCR signaling’s instructive role is evident, species-specific differences in Notch and IL7R signaling add complexity. Crucial transcription factors like EGR1-3, ID3, and SOX13 contribute to γδ lineage determination, accompanied by significant epigenetic modulation.^171^ However, challenges and species-specific variations highlight the ongoing need for deeper research into human γδ T cell development.

## γδ T cell migrate from thymus to periphery or tissue

After undergoing fate determination in the thymus, γδ T cells embark on a remarkable journey to the peripheral tissues, where they establish colonization, particularly in sites such as the skin, mucosa, and intestine.^19^ This intricate process involves a series of tightly regulated mechanisms governed by a multitude of regulatory molecules, signaling pathways, and cellular interactions.^113,172,173^ It is important to note here that the current understanding of γδ T cells from thymus to peripheral organs or circulation primarily relies on research conducted in mice, and there is a lack of extensive evidences in human. Once γδ T cells complete their maturation journey in the thymus, they exit the organ and enter the bloodstream, ready to embark on their migratory adventure. The migration of γδ T cells to specific tissues is orchestrated by a combination of chemotactic signals and adhesion molecules that guide them to their intended destinations.

In the context of skin colonization, the attraction of γδ T cells is mediated by chemokines produced by resident cells in the skin, most notably keratinocytes. These chemokines, including CCL20 (MIP-3α) and CCL27 (CTACK), act as potent chemoattractants for γδ T cells expressing specific chemokine receptors such as CCR6 and CCR10.^174–178^ The interaction between these chemokines and their corresponding receptors prompts the migration of γδ T cells towards the epidermal layer of the skin where they self-renew, allowing them to establish a resident population within the tissue.^113,179,180^ Similarly, the colonization of mucosal tissues, such as the respiratory and gastrointestinal tracts, involves a similar set of chemotactic cues. Epithelial cells lining the mucosal surfaces play a crucial role by producing specific chemokines, such as CCL20 and CXCL16, which serve as attractants for γδ T cells expressing the corresponding chemokine receptors.^181^ For instance, CCR6 and CXCR6 are expressed on γδ T cells and facilitate their migration towards mucosal tissues. These precise chemokine-receptor interactions are pivotal for the directed migration and successful colonization of γδ T cells in these particular tissue microenvironments.^173,182^ As for intestinal colonization, additional factors come into play. The gut-associated lymphoid tissue (GALT), present in the intestinal mucosa, creates a supportive environment for γδ T cell colonization. Within the GALT, specialized cells such as DCs and macrophages present antigens to γδ T cells, influencing their localization and activation within the intestinal tissue. Moreover, the intestinal epithelial cells produce various regulatory molecules, including cytokines and chemokines, which shape the migration patterns of γδ T cells in the gut. These signals, such as TGF-β, IL-15, and IL-7, contribute to the positioning and retention of γδ T cells within the intestinal tissue.^113,173,181,183,184^

During the process of positioning, migration, and colonization in specific tissues, certain signaling pathways play a critical role in guiding γδ T cells to navigate towards their desired tissue compartments. Adhesion molecules may participate in the adhesion and transmigration of γδ T cells across endothelial barriers during tissue homing. Selectins, integrins, and their corresponding ligands on γδ T cells and endothelial cells facilitate the rolling, firm adhesion, and subsequent diapedesis of γδ T cells into the peripheral tissues.^173,185^ These adhesion molecules provide the necessary interactions for the precise localization of γδ T cells within specific tissue microenvironments. Therefore, the migration and colonization of γδ T cells in peripheral tissues are complex processes regulated by a variety of chemotactic signals, adhesion molecules, and signaling pathways. The precise interplay between these factors guides γδ T cells towards their intended tissue destinations, such as the skin, mucosa, and intestine.^10^ Further investigation of these mechanisms in human will advance our understanding of γδ T cells in tissue-specific immune surveillance and responses, further enhancing the potential applications of γδ T cells in disease immunotherapy.

Collectively, during the process of migration and homing to various locations, diverse chemokine receptors on γδ T cells play a critical role in determining whether these cells circulate or become tissue-resident. Although existing insights into the function of chemokine receptors in γδ T cell migration are largely derived from gene-targeted knockout mouse models, such as the CCR9/CCL25 pathway guiding murine γδ T cells to the small intestine,^186^ it is reasonable to hypothesize a similar molecular mechanism in humans. Excitingly, recent research has turned its attention to the homing properties of human γδ T cells, with a specific focus on examining the functional significance of chemokine receptor expression in both healthy individuals and patients.^187^ In the peripheral blood, the predominant Vδ2 subset expresses CCR5, which serves as a receptor for CCL3 (MIP-1α), CCL4 (MIP-1β), and CCL5 (RANTES). Additionally, Vδ2 T cells express CXCR3, the receptor for CXCL10/CXCL11.^188^ CCR5 and CXCR3 are linked to Th1 cells, renowned for their cytokine production, including IFN-γ and TNF-α, upon activation.^189^ In contrast, the Vδ1 subset of peripheral blood γδ T cells demonstrates a distinct preference for CXCR1, the receptor for CXCL5/CXCL6/CXCL8.^188,190^ Notably, Vδ1 T cells, unlike their Vδ2 counterparts, express CCR2 and exhibit migratory responses to CCL2. Significantly altered expression of this chemokine is observed in various human tumors like lung, prostate, liver, or breast cancer.^191^ This divergence in chemokine receptor expression between Vδ1 and Vδ2 T cells underscores distinct homing mechanisms within tumors, suggesting chemotactic properties of γδ T cells are crucial for determining their effectiveness in immunotherapy.

In summary, after thymic fate determination, γδ T cells navigate from thymus to peripheral tissues, including skin, mucosa, and intestine. Chemotactic signals and adhesion molecules orchestrate this journey. In humans, chemokine receptors (CCR5, CXCR3, CXCR1) on γδ T cells demonstrate tissue-specific homing. By probing chemokine receptor profiles, we will be able to unlock insights into cancer immunotherapy with γδ T cell subsets (Vδ1, Vδ2) and their potential for selective targeting. Advances in understanding tissue-specific immune response help refine γδ T cell-based therapies clinically.

## γδ T cell fate from embryo to adulthood and old age

The comprehensive developmental pathway of γδ T cells, spanning from early embryonic phases to adulthood in humans, remains incompletely elucidated. However, a wealth of available data is progressively unraveling the intricacies of this trajectory. Meanwhile, insights gleaned from murine studies also offer invaluable knowledge to infer and construct a plausible framework for the actual progression in humans. During murine embryonic development, γδTCR expression was detected by 14 days of gestation in murine^100^. In human, the Vγ9 and Vδ2 variable (V) gene segments are the first to undergo rearrangement in γ/δ T cell development, and detectable at 5 to 6 weeks of gestation in the fetal liver^192^ and after 8 weeks in thymus^116^. At the mid-gestation (20-30 weeks), Vδ2 T cells become the predominant in the γδ T cell repertoire and is capable of producing IFN-γ^193–195^. However, as gestation progresses, there is an increase in the generation of Vδ1^+^ T cells, which ultimately make up the majority of the γδ repertoire in cord blood and the pediatric thymus.^115,193,196,197^ Therefore, Vδ2 T cells constitutes smaller proportion comparing with Vδ1^+^ T cells at birth.^95,194,195^ Nevertheless, there is a consensus that Vδ2 T cells undergo phenotypic maturation soon after birth^194,198^. Overall, γδ T cells are known to play vital protective roles throughout the lifespan, particularly in defense against infections and transformations. Their early maturation and functional development contribute to their ability to mount effective immune responses and provide immune surveillance against various pathogens and pathological processes.

Once γδ T cells have completed their maturation in the thymus and migrated into peripheral tissues, they embark on the process of aging. Although research on γδ T cells is limited, similar mechanisms observed in αβT cells may also apply to γδ T cells. We thus proposed that epigenetic regulation (e.g. DNA methylation, histone modifications)^199,200^ may play a pivotal role in the aging or exhaustion process of γδ T cells, contributing to their functional decline and altered immune responses. In aged T cells, global DNA hypomethylation and regional hypermethylation have been observed, affecting gene expression and cellular function.^201–203^ Alterations in the balance of histone acetylation and deacetylation, mediated by histone acetyltransferases and histone deacetylases, respectively, can impact T cell function, immune responses, and gene expression patterns. Additionally, specific microRNAs and long non-coding RNAs exhibit altered expression in aged T cells,^204–207^ influencing T cell differentiation, proliferation, and immune signaling pathways by targeting key genes involved in T cell function.^200^

The fate of γδ T cells during aging is also influenced by thymic involution, a gradual reduction in thymus size and output, leading to decreased production of new γδ T cells. This causes gradual reduction in the proportion of peripheral γδ T cells, particularly Vδ2 T cells, from childhood to adulthood and into old age.^194,208,209^ Consequently, the aging microenvironment, characterized by twelve hallmarks of aging,^210^ including changes in cytokine profiles and tissue-specific alterations, affects the localization and function of γδ T cells within tissues. The functional properties of γδ T cells also undergo changes with advancing age, including a decline in proliferative capacity, impaired cytokine production (e.g., IFN-γ and TNF-α), and alterations in receptor expression and signaling molecules.^195,200^

**Fig. 3:**
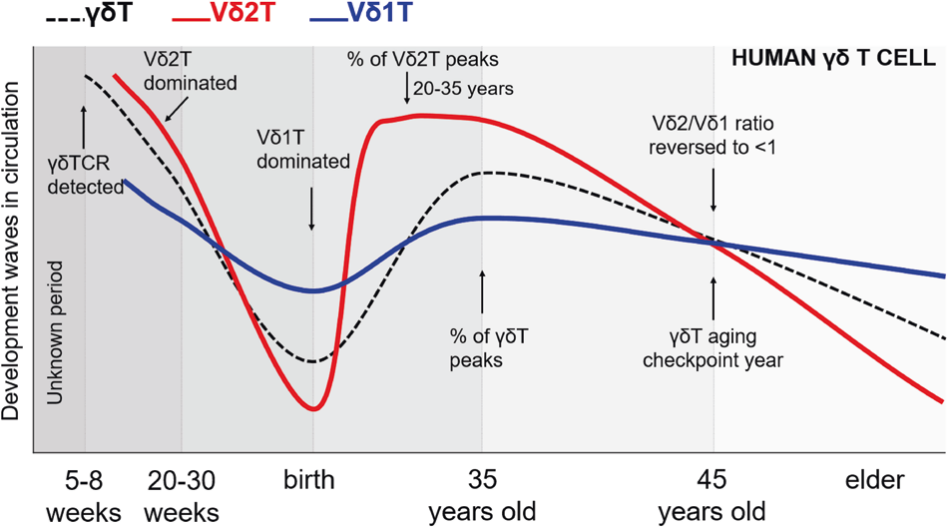
Development waves of two major human γδ T cell subsets in circulation, inspired by models of literatures.^95,242,746,747^ This sketch depicts the variations in total γδT, Vδ2T, and Vδ1T cell populations from embryonic stages through adulthood and into old age. This representation is informed by the integration of our recently published data on γδ T cells, encompassing a cohort of 43,096 healthy individuals spanning an age range of 20 to 88 years^209^

Moreover, following a thorough review of relevant literature models, we have succinctly outlined the developmental trends of circulating γδ T cells across the lifespan, ranging from embryonic stages to advanced age, as visually depicted in Fig. 3. Notably, the identification of γ/δTCR expression occurring around 5 to 8 weeks of gestation in the fetal liver and thymus has been reported,^116,192^ and subsequently, there is a noticeable shift in the predominant population, with Vδ2 T cells assuming dominance during mid-gestation (20-30 weeks) followed by a transition to Vδ1 T cell predominance at birth.^95,193–195^ Significantly, our most recent investigation^209^ unveils that the proportion of γδ T cells within the CD3^+^ T cell population reaches its zenith at 35 years of age. In this context, the proportion of Vδ2 T cells in the overall γδ T cell reaches a plateau within the age range of 20 to 35 years. Yet, the precise age at which the proportion of Vδ2 T cells surpasses that of Vδ1 T cells remains an enigma. Equally noteworthy, our research identifies the pivotal year of 45 as a checkpoint for γδ T cell aging. It is at this juncture that the Vδ2/Vδ1 ratio descends below 1, marking an association with immune aging and characterized by the hallmark of a reversed Vδ2/Vδ1 ratio. This discovery holds profound implications for our understanding of the aging immune system. Altogether, these age-related changes collectively affect the ability of γδ T cells to mount effective immune responses, immune surveillance, tissue homeostasis, and overall immune function.

## γδ T cell subsets

Before delving into the discussion of γδ T cell subsets, it is essential to provide a brief overview of the fundamental knowledge regarding γδ T cells and the diversity of the γδTCR repertoire. The γδ T cells account for approximately 1–5% of total T cells in peripheral blood but much higher proportions are present in various human tissues such as the intestine (nearly 40%^211^) and skin (10–30%^173,183,212,213^). γδ T cells represent a unique subset of lymphoid cells that exhibit characteristics of both innate and adaptive immunity.^83,183,214,215^ Additionally, they are regarded as professional APCs capable of regulating their αβ counterparts.^17,56,216,217^ Furthermore, they can exhibit the function as a “signal processing hub,” receiving signals from and transmitting signals to other immune cells,^16,218^ such as B cells,^219–222^ dendritic cells,^223–226^ macrophages,^227–230^ NK cells,^231–233^ and αβ T cells^56,234,235^ making them an integral part of both innate and adaptive immunity.

Unlike T cells with αβ TCR, the antigen recognition of γδ T cells does not depend on the processing by APCs and subsequent presentation by MHC molecules; thus, they are considered non-MHC restricted.^8,236,237^ This feature of γδ T cells allows them to carry out unique functions compared to their αβ counterparts, resulting in a broader range of immune responses and broader protection. Although MHC-restricted γδ T cells have been discovered, they only constitute a small fraction of the γδ T cell population.^238,239^

Additionally, prior to focusing on the discourse concerning human γδ T cell subsets, we have provided a brief overview of the disparities in γδ T cell profiles between humans and mice. This includes distinctions in the γ/δ chains and their combinations, as well as disparities in distribution, thymic development and antigen recognition. This overview aims to provide readers with a straightforward understanding of the unique attributes of species-specific γδ T cells and subsets (Table 1).

**Table 1:** An overview of the differences in γδ T cell profiles between humans and mice (based on the Tonegawa nomenclature^715^)

Mouse (chains)		Distribution	Development in thymus	Recognition/antigen	Notes	Human (chains)		Distribution	Development in thymus	Recognition/antigen	Notes
Vγ	Vδ					Vδ	Vγ				
Vγ1	Vδ5; Vδ6.3	Thymus, spleen, liver^716^	Emerging in neonatal thymus, prevailing postnatally^717,718^	Mycobacterial Hsp60^36,719,720^	/	Vδ1	Vγ2;Vγ3 Vγ4;Vγ5 Vγ8;Vγ9	Epithelia, dermis, spleen, liver, rare in blood^721^	Mid-gestation onwards^198^	MICA/B^267,268^, CD1c/d^722,723^,^[254^ Lipohexapeptides^724,725^	Paired with diverse Vγ chains^721^
Vγ2	/	Rare	Present in a minority of postnatal thymic γδ T cells^726^	MHC class II gene molecule I-E^k^^727^	/	Vδ2	Vγ9	Peripheral blood^728^	Detectable 5 to 6 weeks in fetal liver^192^	Phosphoantigens^729^, staphylococcal enterotoxin A^730,731^	Vδ2/Vγ9 exclusively pairs^728^
Vγ4	Vδ1 Vδ4; Vδ5; Vδ6; Vδ7	Blood, spleen, lung, lymph nodes^726^	Emerging postnatally and then dominate thymic γδ T cells^717,718^	Diverse gut pathogens^732^, bacterial pathogens^66^, imiquimod^436^	Major γδT cell population in adult thymus, lymph nodes and spleen^733^	Vδ3	Vγ2; Vγ3	Liver, gut epithelium, rare in blood^734,735^	Predominant in late-fetal and neonatal blood^736^	CD1d^737^	Account for ∼0.2% of circulating T cells and respond to CD1d^737^
Vγ5	Vδ1	Epidermis^733,738^	Earliest T cells in murine fetal thymus at day 14^100^	Stressed epithelial cells^739^	Mainly γ chain in intestinal intraepithelial lymphocytes^740,741^	Vδ5	Vγ4	/		Endothelial protein C receptor (EPCR)^656^	Recognizing transformed cells via binding to endothelial protein C receptor.^656^
Vγ6	Vδ1	Uterus,vagina, tongue,placenta, testes, lung, kidney^19^	Present in late fetal and newborn thymus^742^	Commensal microbiota^343^	A major proportion of γδ T cells in uterine tissue^19^	Vδ4; Vδ6; Vδ7; Vδ7	/	Peripheral blood of lymphoma patients^255^		/	/
Vγ7	Vδ4; Vδ5; Vδ6	Intestinal mucosa^717^	Thymic independent^741,743,744^	Stressed intestinal epithelial cells^48^	Paired with multiple Vδ chains^745^						

### δTCR chain-based taxonomy of human γδ T cells

In humans, there are three major γδ T cell subsets classified based on their *TRDV* genes, which are referred to as Vδ1^+^, Vδ2^+^, and Vδ3^+^. However, the Vδ2^+^ subset primarily pairs with Vγ9 TCR, making it the predominant γδ T cell population in circulating blood. In comparison with Vδ1^+^ T cells, which are generated in the human thymus a few months after birth, Vγ9Vδ2 cells develop at early stages of fetal development.^123,194^ Therefore, it is fair to speculate that Vγ9Vδ2 cells serve as the first line of defense and form an integral part of innate immunity^9^. On the other hand, the Vγ9-negative Vδ2^+^ subset has been reported to demonstrate properties of adaptive immunity.^128^

Vδ1^+^ cells are mainly located in the gut epithelium, skin, spleen, and liver, and only a small proportion is detectable in circulating blood. Pairings between Vδ1^+^ and Vγ chains are more flexible than the highly conserved Vγ9Vδ2 TCRs. Sequencing evidence strongly indicates that the TCR diversity of Vδ1^+^ cells mainly originates from *TRD* rather than *TRG* repertoires.^128,214^ Furthermore, strong clonotypic focusing of Vδ1^+^ cells is observed in most adults, and it comprises the private Vδ1^+^ T cell population exclusive to each adult.^115,240^ The above clonotyping and viral infection response studies on Vδ1^+^ and Vδ2^+^ cells all indicate that clonally selected Vδ1^+^ T cells exhibit adaptive immune cell characteristics such as “memory-like” features and rapid clonal expansion capacity, whereas semi-invariant Vγ9Vδ2 T cells align more with innate immunity.^214,240,241^

**Fig. 4:**
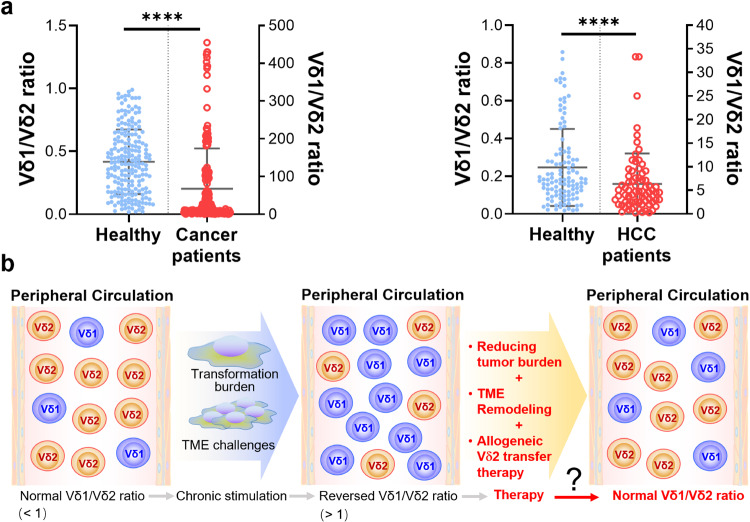
An inverted Vδ1/Vδ2 ratio in the peripheral blood of various solid tumor patients, including those with liver, lung, breast, pancreatic, kidney, and other types of cancer. **a** In healthy populations, the Vδ1/Vδ2 ratio is usually less than 1. However, in cancer patients, including those with hepatocellular carcinoma (HCC), this ratio is reversed, and it becomes far greater than 1 according to our previous work.^243,748^ **b** A hypothetic sketch suggests that the normal Vδ1/Vδ2 ratio is skewed by the burden of transformation and the challenges posed by the tumor microenvironment (TME), resulting in a disordered ratio. Available therapy approaches provide alternatives for re-modulating the TME to achieve the normalization of the Vδ1/Vδ2 ratio and subsequently immune function

Since clonotypic expansions of non-Vγ9Vδ2 T cells (Vδ1^+^, Vγ9^−^Vδ2^+^, Vδ3^+^, etc.) take place in both diseased and healthy individuals, it is presumed that the non-Vγ9Vδ2 TCR repertoires “record” the immunological history (previous antigen challenges) of each individual.^242^ Interestingly, we^243^ and other groups^244–246^ observed an inverted Vδ1/Vδ2 ratio in the peripheral blood and/or tissues in cancers or infectious diseases^187^ (as shown in Fig. 4), this could be explained by the rapid clonal expansion of the “adaptive” Vδ1^+^ subset upon antigen challenges during tumor progression or infections, whereas the “innate” Vγ9Vδ2 population does not thrive under chronic antigen stimulation^247^ and undergoes activation-induced cell death (AICD).^248^ Therefore, a holistic treatment approach encompassing tumor burden reduction, TME remodeling, and the adoptive transfer of allogeneic Vδ2^+^ cells holds promise for re-establishing host immunity and preserving the normal Vδ1/Vδ2 ratio. Nevertheless, further scientific evidence is required to certify this hypothesis.

In contrast to the Vδ1^+^ and Vδ2^+^ subsets, the Vδ3^+^ subset is rarely detected in the peripheral blood of healthy individuals but is enriched in the liver and gut epithelium.^17,193,249–251^ Interestingly, Vδ3^+^ cells recognize similar ligands as the Vδ1^+^ subset.^251^ Moreover, like the Vδ1^+^ subset, they showed an increased expression of CD16 molecules, which are low-affinity IgG Fc region receptors (FcγRIII), and were capable of orchestrating antibody-dependent cellular cytotoxicity (ADCC) in the PBMCs of individuals infected with *Plasmodium falciparum*.^252^ Similar cytotoxic phenotypes or clonal expansion have also been observed in CMV^253^ and hepatitis C Virus infections,^254^ suggesting its role in combating infections. Furthermore, recent studies have demonstrated the infiltration or expansion of this subset in tumors, suggesting its potential role in mediating anti-tumor immune responses.^255–258^ Additionally, in vitro expanded Vδ3^+^ T cells have shown the ability to induce maturation and IgM secretion by B cells.^259^ However, because of its rarity, there is limited evidence available to clearly delineate the functional role of the Vδ3^+^ subset. Therefore, further research is needed to fully unravel the functional role that the Vδ3^+^ subset plays under physiological and pathological conditions.

In this article, we primarily limit our discussion to Vδ1^+^ and Vδ2^+^ (Vγ9Vδ2 subtype unless specified otherwise) T cells, due to their abundance in the literature and experimental/clinical applicability.

#### Vδ1^+^ T cells

Vδ1 T cells are players of adaptive immunity. Vδ1^+^ T cell relies on γδTCR- and natural killer receptors (NKR)-mediated recognition of tumor antigens or stress signals, similar to Vδ2 T cell. However, there are significant differences between the two subtypes. Specifically, Vδ1 TCR recognizes MHC-like proteins of the CD1 family, such as CD1c and CD1d,^260–265^ Annexin A2,^266^ and MHC class I chain-related protein A and B (MICA/B),^267,268^ which are mostly upregulated in transformed cells and virus infected cells. Evidence indicates δ1TCR has a much higher affinity toward CD1d than MICA/B.^262,269^ The drastic difference in their TCR ligand recognition patterns further implies non-redundant roles of Vδ1^+^ and Vδ2^+^ in establishing immune surveillance.^270^ Based on published studies, we can conclude that Vδ1^+^ T cells play a significant role in adaptive immunity among γδ T cell subsets.

Like Vδ2 T cells, Vδ1 T cells also highly express natural killer group 2 member D (NKG2D), which is a stress-sensing molecule that recognizes its cognate ligand MICA/B on the surface of the cancer cells. However, Vδ1^+^ TCR and NKG2D do not share binding sites on MICA/B, and the strength of NKG2D-MICA/B binding is 1000-fold stronger than that of Vδ1^+^TCR-MICA/B.^269^ Despite discrepancies in their antigen recognition, both Vδ2 and Vδ1 T cells rely on secretion of the perforin/granzyme-B mediated secretory and death receptor (TRAIL/TRAIL-R, Fas/FasL) pathways to execute their anti-tumor cytotoxic activity.

#### Vδ2^+^ T cells

Overall, activation and recognition of Vδ2 T cells are dependent on phosphoantigen presence. The ligand recognition by Vγ9Vδ2 T cells mainly falls into two groups, namely γδ TCR-mediated and NKR-mediated ones.^8^ Although γδ T cells were discovered almost four decades ago, knowledge of the exact molecular mechanism of antigen recognition by γδTCR is still rather limited, partly due to the low binding affinity to its ligands, which makes ligand identification difficult.^271^ Different from other γδ T subsets, Vδ2^+^ TCRs recognize phosphoantigens that accumulated in tumor cells due to their dysregulated mevalonate pathway.^272–275^ Notably, phosphoantigens do not directly bind to γδTCR, instead, they bind to the intracellular B30.2 domain of the butyrophilin family protein, BTN3A1.^82,276^ This binding then triggers a conformational change of BTN3A1, allowing its collaborator BTN2A1 to hinge onto the Vγ9 chain of the γδTCR, which then activates Vδ2 T cells.^277–280^ However, whether the Vδ2 chain of the Vγ9Vδ2 TCR is involved in the antigen recognition process is still elusive. In addition to the BTN3A1/BTN2A1-mediated phosphoantigen recognition, Vγ9Vδ2 TCR could also interact with the F1-ATPase, apolipoprotein A-1, or hMSH2, which are often abnormally upregulated in cancerous cells.^281–283^ Interestingly, rodents do not have a homologous γδTCR which can be activated by phosphoantigens. As a consequence, conventional mouse models are not suited to study the significance of phosphantigen-reactive γδ T cells in the context of cancer and infection. The recent discovery of a phosphoantigen-reactive Vγ9Vδ2 TCR in alpacas (Vicugna pacos) has established them as the first non-primate species with this feature.^284^ This introduces a novel model for Vγ9Vδ2 T-related research, complementing the existing nonhuman primate models.

Apart from TCR-mediated antigen recognition, NKR plays crucial roles in activating Vδ2 T cells and initiating tumor lysis. Specifically, NKG2D on Vδ2 T cells binds to MICA/B^285–287^ and UL16 Binding Proteins (ULBPs) of cancer cells,^288,289^ and the DNAX Accessory Molecule 1(DNAM1) on Vδ2^+^ binds to Nectin-like 5 of cancer cells, leading to perforin-granzyme axis mediated cancer cytotoxicity.^290^ Like NK cells, Vδ2 T cells also express CD16 and are capable of orchestrating ADCC upon binding to tumor-specific antibodies.^291–293^ Interestingly, this type of killing appears to be restricted to the Vδ2^+^ subtype but not Vδ1^+^ in an in vitro study.^294^ Conversely, it has been demonstrated that in patients with viral infections, in vivo expression of CD16 on Vδ1 T cells occurs.^252,253^ Therefore, understanding the differences in phenotypic characteristics and the underlying molecular mechanisms between the two subtypes helps in extrapolating their respective clinical advantages.

### Effector subsets defined by cytokine release

The anti-tumor role of γδ T cells was first established by the seminal work of Hayday and his colleagues using TCRδ-deficient mice.^53^ Early studies suggested that γδ T cells serve as an early source of IFNγ and contribute to anti-tumor responses in various cancer types.^295–299^ However, recent advancements have unveiled that γδ T cells can also play pro-tumor roles in cancer. For instance, the pro-tumorigenic role of IL17-producing γδ T cells was validated in IL17 knockout mice which showed slower tumor progression in different models of cancers.^76,79,300–303^

Given that the γδTCR chains do not exhibit a distinct functional bias within the tissue microenvironment, they are insufficient for classifying the immune function of γδ T cells. Therefore, alternative approaches have been employed to functionally define subsets of γδ T cells based on their immune response functions, particularly their ability to release cytokines. Two major effector subsets of γδ T cells can be categorized based on their ability to produce specific cytokines. γδT1 cells, which produce IFN-γ (IFN-γ^+^γδ T cells), mainly playing anti-tumor function. γδT17 cells, which produce IL-17 (IL-17^+^γδ T cells), leading to pro-tumor and autoimmune diseases.^11,16,304^ Notably, γδNKT cells, which produce both IL-4 and IFN-γ, have also received increasing attention.^98,112^ About their development in thymus, both IFN-γ-producing subsets (γδT1 and γδNKT) has been shown to rely on strong signals from the TCRs, whereas γδT17 cells have been reported to develop even in the absence of TCR ligand selection.^63,112,162,305^

**Fig. 5:**
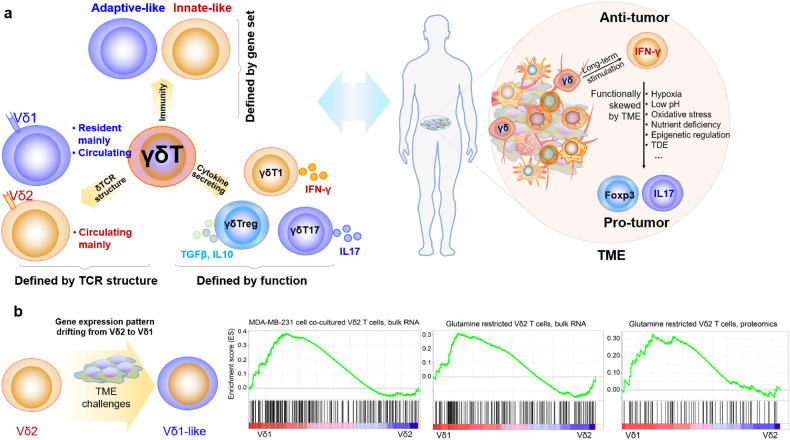
The γδTCR chains are insufficient to classify γδ T immune function. **a** Three ways to subclassify γδ T cells are δTCR structure-based, cytokine secretion-based, or gene expression pattern (or immunity)-based. To our knowledge, in the context of the TME, a function-based γδ T taxonomy would be more objective than the TCR-based approach to describe their pro- or anti-tumor functions, since the immune function tends to be switchable (the right sketch). **b** Interestingly, we observed that, in the context of TME challenges, the gene expression pattern of Vδ2 T cell can be skewed toward that of Vδ1^+^ T cell (the left sketch graph). For example, our group recently discovered that cancer cell coincubation or amino acid (glutamine) stress, which are the common features of the TME, can skew Vδ2 T cells towards a Vδ1-like T cells (at the gene expression level) (the right graph)

Actually, the functional propensities of each subset of γδ T cells are highly context-dependent, as they could be modulated by their immediate environment (as shown in Fig. 5a). Specifically, cytokines produced by γδ T cells under distinct circumstances help to define their functions more precisely. γδT1 mediate intracellular pathogen clearance and elicit anti-tumor immunity, whereas γδT17 provide protection against extracellular bacteria and fungi infections. Another less well-characterized functional subset of γδ T cells that carries out regulatory functions in cancer or inflammatory diseases has been identified as γδTreg.^306–311^ This population is induced upon receiving Inflammatory signals in the TME and could potentially sabotage the anti-tumor phenotype of γδ T cells while reprogramming them into γδTreg.^306,307,312^ This subset has been identified as CD73^+^Foxp3^+^Vδ1^+^ T cell in the PBMC or tumor specimen of breast cancer patients^313^ and tumor-infiltrated CD39^+^Foxp3^+^γδT in colon cancer. Both CD39^+^ and CD73^+^ γδTreg possess immune-regulatory functions.^314,315^ Lastly, a minor subtype of γδ T cells that could initiate a Th2-like response (IL-4 production) under pathological conditions has been identified.^43,316^ The above evidence further supports the functional plasticity of γδ T cells is context-dependent.^8,17,317,318^

Furthermore, accumulating evidence reveals that the immune function of both Vδ1^+^ (generally pro-tumor) and Vδ2^+^ (generally anti-tumor) subsets is plastic and depends on the specific cytokine milieu. Vδ2 T cells could be skewed toward IL17-producing γδT17 when stimulated with a cytokine cocktail of IL-1-β, TGF-β, IL-6, and IL-23 in vitro,^81^ and they can also be induced into *FOXP3*-expressing Treg in the presence of TGFβ1, IL-15, and antigen stimulation.^319,320^ In the additional presence of the epigenetic modifier Vitamin C, the FOXP3 locus is specifically demethylated, in line with regulatory function.^320^ An early study showed that IL4 could negatively impact γδ T cell-mediated tumor immunity, skewing γδ T cell population toward the IL-10-secreting Vδ1^+^ instead of the IFNγ-secreting Vδ2^+^ subset.^312^ Clinically, both IL17-producing Vδ2^+^ and the IFNγ-producing Vδ1 T cells have been found in cancers,^321–323^ and distinctive cytotoxic hallmark patterns were found on Vδ1^+^ and Vδ2 T cell subsets, respectively.^324^ Moreover, intrahepatic γδ T cells are mainly comprised of polyclonal Vδ1^+^ subsets that are phenotypically distinct from those in the matching blood, implying functional plasticity of the Vδ1^+^ T cells.^241^ Importantly, Hayday’s group correlated Vδ1^+^ but not Vδ2 T cells with better outcomes in the patient with triple-negative breast cancer (TNBC), suggesting a protective role of a subset of Vδ1^+^ T cells.^325^ By analyzing RNA sequencing data, we observed a shift from Vδ2^+^ to Vδ1^+^ subset gene expression profiles in in vitro expanded Vδ2^+^ cells after co-culturing with MDA-MB-231 TNBC cell line. A similar shift was also observed when Vδ2^+^ cells were cultured under the glutamine (one of the main nutrients deprived in TME) deficient condition (Fig. 5b). These phenotypes indicate the plasticity of Vδ2 T cells, once again demonstrating that a TCR-based classification is insufficient to describe the functional signatures of γδ T cells in the TME. Therefore, one cannot simply classify Vδ1^+^ and Vδ2^+^ subsets’ functions based on their respective TCR signatures, since the properties of γδ T cells in tumorigenesis may be pleiotropic depending on the tumor type and stages.^9,321^

Additionally, beyond tumors, distinct functional heterogeneity and plasticity have been observed among γδ T cell subsets, which can play either protective^60,84,326,327^ or detrimental^328–330^ roles in the context of infections and autoimmune diseases. Hence, a thorough understanding of the intricate functional behaviors and phenotypic variations of γδ T cell subsets is crucial to elucidate their roles in diverse disease contexts. Therefore, in the subsequent subsections, we proceeded to provide a comprehensive discussion on IFNγ-producing γδ T (γδT1), IL-17-producing γδ T (γδT17), regulatory γδ T (γδ Treg), and antigen presenting γδ T cells (γδ T_APC_).

#### IFNγ-producing γδ T (γδT1): anti-tumor role and plasticity

An infiltrated or circulatory IFNγ-producing γδ1^+^ T cell population has been considered a positive prognostic marker in cancers.^8,297,307^ For instance, Dieli’s group observed a positive correlation between the frequency of γδ TILs in the tumor specimen and the 5-year patient prognosis in 557 colorectal cancer (CRC) patients.^331^ However, this conclusion was challenged by evidence indicating that proinflammatory γδ17 may contribute to cancer development in various tumor models.^80^ Similarly, immunosuppressive γδTreg has been found to positively correlate with the progression of CRC^314^ and breast cancer.^332^ A recent discovery has also shown that the conversion of IFNγ-producing γδ T to IL17-producing ones occurs as CRC progresses,^333^ underscoring their functional plasticity shaped by the TME.^334^ Furthermore, it is still unclear whether tumor-infiltrated γδ T cells come from the original tissue-resident ones (characterized with surface markers CD69 and CD103^335–337^) or peripheral blood, or both.^14^ Hence, elucidating the functional diversity and plasticity of γδ T cells across various cancer types is necessary. Using the ‘deep deconvolution’ CIBERSORT algorithm,^338^ Gentles et al. conducted extensive transcriptomic analyses on tumor biopsy samples across 39 cancer types with over 18,000 samples and concluded that infiltrated-γδ T cell is the best prognostic immune cell subset (out of 22) to predict favorable patient outcomes.^86^ However, a follow-up study with an optimized deconvolution strategy separating γδ T cells from NK and αβ T cells contested this conclusion, suggesting a much looser correlation between γδ TIL and cancer prognoses in 50 hematological and solid malignancies.^339^ Therefore, the application of spatiotemporal scRNA-seq or single-cell proteomics can enable the in-situ clarification of the functional contributions of individual γδ T subsets (γδ1, γδ17, and γδTreg, etc.) and their functional evolution in the TME. Recently, we carried out functional phenotyping of γδTILs of HCC patients by scRNA-seq and found low *IL17A* but high *IFNG* expression in γδTILs (mostly Vδ1^+^), implying cytotoxic effector function of γδTILs in HCC.^243^ Since γδ T cells display heterogeneity across cancer types or even among individuals, more sophisticated and thorough studies are needed to truly shed light on the functional discrepancies and plasticity of γδ T cells and facilitate their clinical applications. Moreover, deciphering the molecular mechanisms underlying the spatial and temporal functional pleiotropy of γδ T cells, specifically the signature effector functionalities of individual subsets, can help develop intervention strategies to skew the function of γδ T cells in cancer patients towards an anti-tumorigenic effect.

#### IL-17-producing γδ T (γδT17): pro-tumor and pro-inflammatory role

Different from mice, the IL17-producing γδT17 population is scarcely found in healthy individuals but undergoes rapid expansion in proinflammatory milieu such as acute infections^81^ and cancers.^80,321,340–342^ The evidence indicates that circulating and/or tissue-resident γδT17 cells promote the metastasis of breast tumors,^302^ the progression of liver cancer^79^ and lung cancer,^343^ and are associated with poorer prognoses in patients with colon^80^ and gall-bladder cancers.^340^ IL-1β, an inflammatory cytokine secreted by myeloid lineage cells in the TME, has been found to skew γδ T functional polarization toward γδT17 subtype in various cancer models.^78,301,302^ Importantly, a randomized, double-blinded trial on 10,061 patients, dubbed as “CANTOS” study, demonstrated IL-1β antibody inhibition could greatly decrease both the incidence and mortality rate of lung cancer.^344^ This evidence further supports the pro-tumorigenic functions of γδT17. Moreover, IL17-mediated interactions between γδ T and myeloid lineage cells facilitate cancer progression. For instance, γδT17 recruits immunosuppressive myeloid-derived suppressor cells (MDSCs) into the TME.^76,79,80^ A recent study even demonstrated that commensal microbiota could promote IL17 secretion in lung-resident γδ T cells, which then promote tumor progression.^343^ Interestingly, evidence indicates that the presence of γδT17 is essential for the efficacy of chemotherapy by facilitating the recruitment of IFNγ-producing cytotoxic CD8^+^ TILs.^345^ Therefore, further evidence is required to elaborate the role(s) of γδT17 in cancers.

γδT17 cells are involved in both proinflammatory diseases and infections. They contribute to tissue inflammation and immune dysregulation in conditions like autoimmune disorders.^7,20^ In infections, they actively participate in pathogen clearance by producing IL-17, IFN-γ, and other proinflammatory cytokines, while activating immune cells such as macrophages and neutrophils.^57,59,346^ However, dysregulated activation of γδT17 cells can lead to tissue damage^71,347^ and chronic inflammation,^309^ even autoimmune diseases like psoriasis.^69,313,348^ Understanding their intricate regulation network is important for developing effective treatment regiments.

In conclusion, gaining further insights into the thymic development process and the diverse array of factors within the immediate microenvironment surrounding γδ T cells is essential for a comprehensive understanding of the functional evolution and plasticity exhibited by distinct subsets of γδ T cells, whether characterized by their TCR chains or the cytokines they release, as discussed earlier. This enhanced understanding has the potential to significantly improve our interpretation of the roles γδ T cells play in both normal physiological processes and pathological conditions. Consequently, it can aid in the development of more effective immunotherapies based on harnessing the potential of γδ T cells.

#### Unveiling novel effector functions: regulatory (γδTreg) and antigen-presenting (γδT_APC_) roles

Accumulating evidence has unveiled the multifaceted roles of γδ T cells in humans, extending beyond their roles in anti-/pro-tumor or anti-/pro-inflammation responses. They also exhibit crucial functions as regulatory immune cells known as γδTreg and as γδT_APC_ involved in the process of antigen recognition. Notably, emerging research suggests that effector γδ T cells can transition into γδTreg under specific microenvironmental conditions.^306–311^ Previously, we had thoroughly reviewed the regulatory functions of γδ T cells,^349,350^ particularly Vδ1 and Vδ2 subsets, it was demonstrated that these subsets can be induced to express FoxP3 and execute regulatory functions in the presence of TGF-β, IL-2, and IL-15.^351^ Similar to conventional Tregs, human γδ T cells employ various molecules such as GM-CSF, IL-10, TGF-β, IL-17, CD39, CD73, and checkpoint receptors as part of their immunosuppressive mechanisms.^350^ Notably, the Vδ1 subset, majorly tissue-resident, displays a propensity to convert into γδTregs, as indicated by the expression of CD73^+^ and CD39^+^ phenotypes in cancer patients, although consistent Foxp3 expression has not been universally observed.^313–315^ Our research (Fig. 5b), alongside reported literatures,^319,352^ supports the notion that Vδ2 T cells can also be skewed towards γδTreg under specific microenvironmental cues, such as the presence of TGFβ1, IL-15, and antigen stimulation. Remarkably, Vitamin C has been identified as a catalyst for the conversion of Vδ2 T cells into Foxp3^+^γδTreg.^320^ Taken together, above work underlines the substantial functional plasticity of γδ T cell subsets, with their effector functions subject to modulation by microenvironmental factors.

On a separate note, a distinctive feature of human γδ T cells, notably the Vδ2 subset, is their capability to serve as professional APC to transmit antigen signals to αβT cells, including CD4^+^ and CD8^+^ T cells. The antigen-presenting function of Vδ2 T cells was initially reported by Brandes in 2005,^56^ emphasizing the immunological importance of Vδ2 T cells in adaptive immunity regulation. Subsequent studies proposed that the APC function of human blood-derived γδ T cells is precisely regulated spatially and temporally, requiring pre-sensitization with specific antibody-coated target cells for full APC functionality.^353^ Furthermore, it was demonstrated that the APC function of γδ T cells can be compromised in conditions such as sepsis, resulting in impaired activation of CD4^+^ T cells. Conversely, γδ T cells from healthy individuals retain normal APC function.^354^ This observation aligns with our findings indicating that allogeneic Vδ2 T cells from healthy donors demonstrate promising clinical effectiveness in solid tumor patients.^11,12^ Our research also indicated that the infusion of allogeneic Vδ2 T cells can increase the proportions of CD4^+^ and CD8^+^ T cells in the blood of most patients (refer to Fig. 7d), consistent with the APC function of Vδ2 T cells, which can promote αβT cell proliferation.^354^ It is this APC function that positions the adoptive transfer of Vδ2 T cells as a promising strategy for tumor immunotherapy. Therefore, the exploration of how to effectively exploit the potential of Vδ2 T cells for the utmost benefit of patients requires further investigation. Specifically, a deeper understanding of the underlying molecular regulatory mechanisms of γδT_APC_ is imperative.

## γδ T cell and diseases

**Fig. 6:**
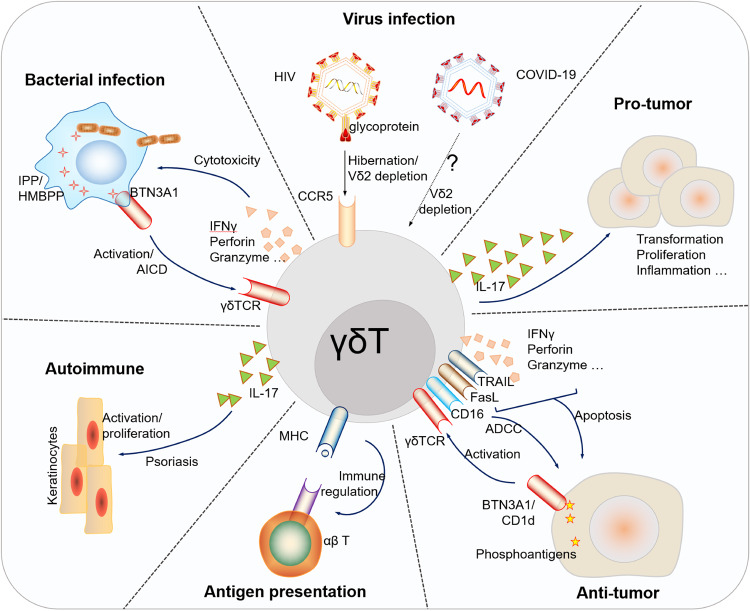
Brief sketch depicts the major roles of human γδ T cells in the immune regulation, pathogenesis and progression of diverse diseases (representative mechanisms shown). AICD activation induced cell death, ADCC antibody-dependent cellular cytotoxicity, HMBPP (*E*)-4-Hydroxy-3-methyl-but-2-enyl pyrophosphate, IPP Isopentenyl pyrophosphate; The ‘?’ means the molecular mechanism is not clear yet

Accumulating evidence now strongly affirms the multifaceted role of γδ T cells in the pathogenesis and progression of a multitude of diseases. This encompasses infections initiated by pathogens such as viruses and bacteria, autoimmune disorders, tumor, and more. To begin, we provide a brief overview of the contributions of γδ T cells to these diseases, including their function as APCs, as depicted in Fig. 6.

### γδ T cell in infectious diseases

γδ T cells play protective roles in infectious diseases. Unlike conventional αβ T cells, which recognize peptide antigens presented by MHC molecules on APCs, γδ T cells have unique TCRs that allows them to recognize a wide variety of peptide or non-peptide antigens, including microbial products, stress-induced molecules, and self-antigens. Once activated, γδ T cells initiate a rapid immune response against pathogens by directly recognizing conserved molecular patterns expressed by various microbes, such as lipopolysaccharides (LPS), lipoteichoic acid (LTA), *via* pattern recognition receptors, and phosphoantigens *via* the TCR. Afterward, activated γδ T cells exhibit cytotoxic capabilities and directly eliminate infected cells by releasing cytotoxic molecules, such as perforin and granzymes, which induce apoptosis in target cells. This cytotoxicity is particularly important for controlling intracellular pathogens, including viruses and certain intracellular bacteria. Furthermore, γδ T cells are potent producers of various anti-infection cytokines, including IFN-γ, IL-17, and IL-22. These cytokines play key roles in recruiting and activating other immune cells, such as neutrophils, dendritic cells, macrophages, and NK cells, to eliminate pathogens and promote tissue repair. Additionally, γδ T cells interact with other immune cells, including αβ T cells, B cells, and NK cells, through the secretion of modulatory cytokines or direct cell-to-cell contact. These interactions help shape the intricate immune network and optimize the innate and adaptive immune responses against pathogens, facilitating their rapid clearance.

#### γδ T cells in *M.tb* infection

TB is a highly contagious airborne disease caused by the *M.tb* infection. According to the “Global Tuberculosis Report 2022” by the World Health Organization (WHO), TB is the leading cause of death globally attributed to a single infectious bacterium, second only to COVID-19.^355^ The progression of TB heavily depends on the ability of *M.tb* to evade and manipulate the host immune responses.^356–359^ TB could evade host immune surveillance and exploit host macrophages and other immune cells, aiding its evolution within the human host.^360–363^ Early studies have shown peripheral expansion of γδ T cells following TB infection^364^ and demonstrated that resident pulmonary lymphocytes express high levels of γδTCR, suggesting their crucial role in fighting against TB infection at the frontline.^40,246,365,366^ Additionally, high-throughput immune repertoire sequencing has the potential to provide fresh insights into the roles of γδ T cells in TB,^246^ including the identification of new *M.tb* proteins as potential ligands that bind to γδTCR, thereby activating γδ T cell-mediated immunity.^367^ Moreover, γδ T cell could recognize a wide range of non-peptidic antigen such as phospho- and lipid-antigens, maximizing its protective role against *M.tb* infection.^368–370^ It has been shown that both IFN-γ and IL-17A/IL-17F-mediated immunity are crucial for γδ T cells to fulfill their roles in curbing *Mycobacterium* pathogenesis.^371–375^

Interestingly, the Vγ9Vδ2 T cell subset but not others expands shortly after birth and exhibits potent cytotoxic functions, serving as a protective mechanism against sudden microbial exposure such as *M.tb* in newborns.^376^ Early studies have indicated the presence of memory-like responses in Vδ2 T cells following Bacille Calmette-Guérin (BCG) vaccination. Given that BCG is a mycobacterial strain like *M.tb*, it is speculated that TB infection could elicit similar immune responses in Vδ2 T cells.^377^ Therefore, Chen and colleagues further investigated the adaptive immune response of γδ T cells in TB-infected primates, suggesting that immunizing Vδ2 T cells could be a promising strategy for TB vaccine development.^378–383^ Based on these findings, we conducted a groundbreaking clinical trial utilizing allogeneic Vδ2 T cell therapy in the treatment of MDR-TB. The results showed a reduction in *M.tb* load and the healing of pulmonary lesions, indicating an enhancement of the host’s immune defenses.^13^ Furthermore, studies have demonstrated that co-administration of phosphoantigens with IL-2, resulting in the expansion of the Vδ2^+^ subset, improves the treatment outcome of TB in macaques.^384,385^

Recently, a study showcased the expansion of a distinctive subset of NK-like CD8^+^ γδ T cells (predominantly Vδ1^+^) during chronic *M.tb* infection. This subset was found to be functionally and clonally distinct from the well-studied pAg-reactive Vδ2 T cells that expand during acute *Mtb* infection.^386^ Moreover, it has been observed that lung tissue-resident γδ T cells in TB patients primarily consist of the Vδ1^+^ subset, rather than the Vδ2^+^ subset,^246^ which raises the question of whether circulating Vδ2 T cells could infiltrate lung tissue and eliminate *M.tb*-infected cells. Therefore, conducting further research to unravel the mechanisms underlying γδ T cell-mediated immune responses in TB, particularly the functional diversity of each subset in peripheral and local inflammatory sites, could make a significant contribution to the advancement of γδ T cell immunotherapy for TB.

#### γδ T cells in human immunodeficiency virus (HIV) infection

HIV is a retrovirus characterized by its composition of two copies of positive-sense single-stranded RNA. Its primary targets are CD4^+^ T cells, namely helper T cells. HIV attaches to CD4 receptors on the surface of these cells, along with co-receptors such as CCR5 or CXCR4, facilitating its entry into the CD4^+^ T cells. Once inside the CD4^+^ T cell, the viral RNA will be reverse transcribed into DNA and thus integrated into the host cell’s DNA, permanently becoming part of the cell’s genetic material. Following integration, the virus exploits the host cell’s machinery to produce viral proteins and replicate the viral RNA, resulting in the formation of new viral particles. Subsequently, these newly formed viral particles are released from the infected CD4^+^ T cell, capable of infecting other CD4^+^ T cells and various immune cell subsets like DCs and macrophages. This widespread infection and subsequent destruction of immune cells contribute to the progressive deterioration of the patient’s immune system.^387,388^ The cumulative impact of HIV weakens the immune system’s ability to mount effective immune responses, rendering individuals more susceptible to opportunistic infections, such as TB and other complications.^389,390^ Regarding the impact of HIV on the γδ T cell subset, early studies have shown a depletion in the Vδ2 subset, along with an increased level of the Vδ1^+^ subset. As a result, an inverted Vδ1/Vδ2 ratio was detected in HIV infected primates.^46,187,391–395^ Previous study indicated HIV envelope protein gp120 could bind to integrin α4β7 and CCR5 on Vδ2 T cells and activate caspases-dependent apoptosis, ultimately inducing the AICD of Vδ2^+^ subset.^244^ Interestingly, the Vδ1^+^ subset is spared from HIV virus-mediated killing in patients due to its lack of the CCR5 receptor, which is involved in this mechanism.^396^ Furthermore, functional profiling has revealed that γδ T cells in HIV patients with rapid disease progression produce higher levels of IL-17 but not IFNγ. This observation is also positively correlated with γδ T cell activation, indicating the crucial role that γδ17 plays in HIV pathogenesis.^393^ Additionally, the role of γδ1 T cells in controlling HIV virus was demonstrated through the production of IFNγ in HIV-exposed seronegative individuals, highlighting the specific immune response against the HIV Gag peptide. This finding strongly suggests that γδ1 T cells play a crucial role in mediating the immune defense against HIV.^397^

Furthermore, NKp30^+^Vδ1^+^ T produced high levels of CCL3, CCL4, and CCL5 to suppress the replication of HIV-1 within CD4^+^/CCR5^+^ human lymphoid cells.^398^ Vδ1^+^ subset, isolated from the PBMCs of both HIV-1 infected patients and healthy donors, secreted both IFNγ and IL-17 upon stimulation with Candida albicans. On the other hand, Vδ2^+^ subset secreted IFNγ and IL-17 in response to mycobacterial or phosphoantigens. These findings suggest a nonredundant role for these two γδ T subsets in HIV patients, as they play vital roles in fighting against opportunistic infections and partially compensating for the loss of CD4^+^ T cells in HIV-infected patients.^61^ Additionally, there is an increased production of IFN-γ and TNFα in Vδ1^+^, while the reverse is true for Vδ2^+^ in HIV-infected patients. This further suggests the compensating role that Vδ1^+^ might play in rescuing the loss of Vδ2^+^ in these patients.^399^ Given the significant correlation between the loss of Vδ2^+^ cells and HIV progression, there has been considerable interest in exploring methods to restore and enhance the antiviral effector functions of γδ T cells.^400^ Interestingly, the loss of the circulating Vδ2^+^ population and its ability to secrete IFNγ could be restored and closely correlated with the increase in CD4^+^ T cell count in chronic HIV-infected patients who received highly active antiretroviral therapy (HAART). This finding further supports the possibility of utilizing the quantity and quality of Vδ2 T cells as a convenient biomarker to assess the effectiveness of HAART treatment in patients.^401^

Hence, there is a natural inclination to consider the clinical restoration and reconstitution of γδ T cell population in HIV patients as a potential beneficial approach for disease control. Successful in vitro expansion of γδ T cells from HIV^+^ donors was accomplished using zoledronate/IL-2, demonstrating cytotoxic effects towards malignant cells.^402^ Furthermore, a prior study explored the clinical application potential of ex vivo expanded Vδ2 T cells derived from HIV patients, revealing their effectiveness in suppressing virus replication in autologous infected CD4^+^ T cells.^403^ Encouragingly, recent advancements in single-cell transcriptomics on the PBMCs of HIV patients have provided an opportunity to gain a deeper understanding of the functional roles and evolutionary dynamics of γδ T cells in the context of HIV infection.^404,405^ While ongoing research and further clinical trials are necessary, γδ T cell immunotherapy holds great promise as a distinct and innovative approach in the treatment of HIV.^406^

#### γδ T cells in COVID-19 infection

COVID-19 is an infectious disease caused by the severe acute respiratory syndrome coronavirus 2 (SARS-CoV-2) and has rapidly evolved into a global pandemic.^407^ SARS-CoV-2 belongs to the coronavirus genus and is an enveloped, single-stranded ribonucleic acid (RNA) virus.^408,409^ The virus gains entry into the host cell by binding its spike protein (S protein) to the angiotensin-converting enzyme 2 (ACE2) receptor on the surface of host cells. This attachment is followed by membrane fusion, allowing the injection of viral RNA into the host cell. Once inside, the viral RNA takes control of the host cell’s machinery, leading to the production of new viral particles. Subsequently, the virus can infect other cells, contributing to the further spread of the infection. Moreover, SARS-CoV-2 exhibits higher mutation rates in comparison to DNA viruses. Mutations in the viral genome arise during the replication process of the virus and facilitate immune (both innate and adaptive) evasion of this virus.^410–413^ Since SARS-CoV-2 infection can lead to dysregulated immune responses, including an excessive release of pro-inflammatory cytokines, such as IL-6, IL-17, IFNγ, and IL1β, often referred to as a “cytokine storm”.^414–416^ This cytokine dysregulation can impact both innate and adaptive immunity and lead to the dysfunction and exhaustion of immune cells and tissue damage. Several studies demonstrated the impact of COVID 19 on the number and function of γδ T cells in the peripheral blood.^87,89,417–419^ For instance, compared to healthy donors, patients with COVID-19 exhibit a significant decrease in the Vδ2^+^ subset of γδ T cells and an inverted Vδ1/Vδ2 ratio. A comprehensive immune profiling on moderate to severe COVID-19 patients suggested an overall increase in innate immune cells (monocytes, neutrophils, and eosinophils) while a reduction in T cell population.^88,420^ Subsequent functional analysis demonstrated decreased secretion of IFN-γ and elevated secretion of IL-17A, along with increased expression of PD-1, in peripheral γδ T cells of patients. Considering that the Vδ2^+^ subset is the main source of IFN-γ, these findings imply that excessive inflammation in COVID-19 patients could potentially lead to reduced responsiveness or AICD of peripheral Vδ2 T cells and/or their migration towards inflammatory lungs. This notion is further substantiated by the considerably higher levels of IFN-γ observed in tissues compared to blood samples.^89^ The role of Vδ1^+^ T cells in COVID-19 has received limited attention due to its low presence in the peripheral blood. However, Vδ1^+^ is the predominant subset of tissue-associated γδ T cells, known for their swift responses against pathogens. A recent study highlighted the rapid activation and expansion of peripheral Vδ1^+^ T cells in nonhuman primates during SARS-CoV-2 infection. Notably, Vδ1^+^ T cells from both peripheral and Bronchoscopy and Bronchoalveolar Lavage (BAL) fluid were skewed toward IL-17-producing functionality, suggesting viral suppressing and proinflammatory role it plays. This observation is further supported by a positive correlation between the frequency of circulating Vδ1^+^ T cells and the viral load in BAL fluid during the early phase of infection.^421^ Given the known presence of γδ T cells, particularly the Vδ1^+^ subset, in lung tissue and their ability to exhibit distinct physiological or pathological functions based on the local microenvironment,^246,323,422^ their involvement in mediating the clearance or disease progression of COVID-19 is not surprising. These findings collectively indicate the active participation of both Vδ1^+^ and Vδ2^+^ subsets in the control of SARS-CoV-2. Recent advancements in single-cell multi-omics techniques applied to samples from COVID-19 patients have provided valuable data for the further evaluation of the functional and developmental characteristics of both peripheral and tissue-resident γδ T cell populations.^423–425^ These studies have the potential to enhance our understanding of the precise functional evolution, developmental trajectory, and γδTCR clonotypic variations of each γδ T cell subset, both in circulating and local inflammation sites, throughout the course of SARS-CoV-2 infection. In summary, further research is needed to delve deeper into these aspects and gain comprehensive insights into the role of γδ T cells in the context of SARS-CoV-2 infection.

### γδ T cell in autoimmune disease

Unlike conventional αβ T cells, which recognize peptides presented by MHC molecules on APCs, γδ T cells do not rely on MHC presentation for antigen recognition. As a result, γδ T cells have a much wider capacity for antigen recognition and can respond to non-peptide antigens, including stress-induced molecules and microbial elements. Given their broad recognition capabilities, it is reasonable to speculate on the role that γδ T cells may play in the development of autoimmunity. In instances where the immune system becomes dysregulated and mistakenly launches attacks on the body’s own healthy cells and tissues as if they were foreign invaders, the involvement of γδ T cells could be significant.

#### γδ T cells in psoriasis

Psoriasis is a chronic autoimmune skin disorder that affects as much as 2–3% of the world’s population and characterized by the formation of red, inflamed skin patches covered with silvery scales, known as psoriasis plaques. The overproduction of IL-17 in psoriatic lesions is one of the primary factors contributing to the dysregulated immune system that leads to the development of psoriasis.^7,20^ IL-17 functions as a pro-inflammatory cytokine, inducing inflammation and recruiting immune cells in the skin. Additionally, it stimulates the proliferation and activation of keratinocytes, the predominant cell type in the epidermis, leading to the characteristic inflamed, thickened, and scaly appearance of the skin. In addition to CD4^+^ T cells that produce IL-17, γδT17 cells have emerged as another significant source of IL-17, playing a pivotal role in driving the progression of psoriasis.^69,71,73,74^ Mice deficient of *Sox13*, a key transcription factor regulating γδT17 differentiation, led to selective deficiency of γδT17 cells during thymic development and are protected from psoriasis-like dermatitis.^75^ Furthermore, γδT17 cells have been found to exhibit a higher abundance in psoriatic skin lesions compared to healthy skin.^426,427^ These cells possess the ability to release cytokines, such as IL-17 and IFN-γ, which contribute to inflammation and the proliferation of keratinocytes. As a result, this process leads to the formation of the characteristic plaques seen in psoriasis.^179,428^ Multiple immune receptors signaling pathways^348,429–433^ and metabolic enzymes or mediators^313,330,434,435^ have been found to promote the differentiation of γδT17 cells in psoriasis. For instance, our research group discovered that GLS1-mediated glutaminolysis is essential for γδT17 cell differentiation and keratinocyte proliferation, thereby contributing to the pathogenesis of psoriasis.^313^ Additionally, we discovered that mTOR1 and mTOR2 signaling pathways regulate the differentiation of γδT17 and are dysregulated in psoriasis-like mouse model.^434^ Recently, it has been shown that γδT17 cells exhibit dynamic trafficking patterns, moving to and from lymph nodes and sites of skin inflammation.^436,437^

Additionally, a study demonstrated that peripheral but not tissue-resident γδ T cells could regulate neutrophil expansion and recruitment in the pathogenesis of psoriatic arthritis, suggesting the complementary role γδ T cells plays in exacerbating the disease progression.^438^ Conversely, other specialized immune cells also participate in facilitating the differentiation of γδT17 in psoriasis. For instance, certain microbial components, such as mannan, could activate macrophages, leading to the secretion of TNF-α. This, in turn, stimulates local γδ T cells to produce IL-17A.^439^ Moreover, nociceptive sensory neurons establish close contact with dermal DCs and regulate their production of IL-23, which plays a crucial role in the differentiation of dermal γδT17 cells.^440^

Considering the crucial role played by IL-17, produced by both Th17 and γδT17 cells in the pathogenesis of psoriasis, recent therapeutic strategies have primarily focused on reducing IL-17 production, counteracting its effects by corresponding inhibitors or antibodies, or limit the chemotaxis of Th17 and γδT17 cells.^429,441–448^ However, adverse side effects such as neurological diseases, infections, and liver dysfunction have been reported.^449–451^ This may be partly attributed to the significant role IL-17 plays in combating certain pathogens, particularly fungal infections. Blocking IL-17 partially compromises the immune system, increasing the likelihood of opportunistic infections.^451^

Notably, it has been demonstrated that excessive dietary cholesterol exacerbates γδT17-cell-mediated psoriasis.^176^ Furthermore, evidence indicated that feeding mice with a western diet, characterized by high fat and simple sugar content or high fat diet alone can induce psoriasiform dermatitis by promoting the accumulation of dermal γδT17 cells.^452,453^ This suggests that dietary interventions could serve as an alternative approach for controlling psoriasis. Furthermore, the application of scRNA-seq on the skin samples from patients holds the potential to shed light on the role that γδ T cells might play in the etiology and progression of psoriasis, providing valuable insights into their intricate involvement in the disease.^454–457^ Altogether, psoriasis is an autoimmune skin disorder driven by IL-17 overproduction, involving both Th17 and γδT17 cells. These cells induce inflammation and keratinocyte proliferation, contributing to plaques. Therapies targeting IL-17 face challenges due to its dual role in immunity. As our understanding of γδ T cells’ involvement grows, new treatment approaches are emerging for improved psoriasis management.

#### γδ T cells in inflammatory bowel diseases (IBDs)

γδ T cells have been implicated in the pathogenesis of inflammatory bowel disease (IBD) characterized by chronic inflammation of the gastrointestinal (GI) tract, including Crohn’s disease and ulcerative colitis.^10,458^ In the gut mucosa, γδ T cells belong to a group of non-classical intraepithelial lymphocytes (IELs) residing within the intestinal epithelium. γδ IELs are present in higher numbers compared to other tissues and are considered essential regulators of intestinal homeostasis and immune responses.^459,460^ Moreover, their unique anatomical position enables them to act as the first-line defenders against intestinal pathogen invasion.^10^ An increase γδ T cell frequency in the diseased intestinal mucosa has been reported in the IBD patients.^460,461^ γδ T cells actively contribute to a multifaceted immunoregulatory role in coordinating both innate and acquired immune responses, thereby preserving the integrity of epithelial tissues. Early study indicated a protective role γδ IEL plays in a chemical-induced acute colitis model.^462–465^ Moreover, recent study has indicated that γδ IELs could promote the viability of Paneth cells, which locate in the small intestine and are responsible for executing antimicrobial functions in Crohn’s disease.^466^ Notably, an early study demonstrated a decreased frequency of the intestinal CD8^+^ γδ T cell subset (mainly Vδ1^+^) in both the peripheral blood and the gut of patients with IBD. This functionally distinct subset exhibits cytotoxicity and produces IFN-γ and TNF-α instead of IL-17.^467^ On the other hand, γδ T cells have been shown to exacerbate chronic ulcerative colitis.^468^ Moreover, γδ IELs have been found to contribute to the excessive shedding of apoptotic enterocytes into the intestinal lumen, which is characterized in IBDs and is linked with disease reoccurrence.^469^ These findings underscore the intricate interplay between γδ T cells and the immune response in IBDs and highlights the need for further research to uncover their precise mechanisms.

Emerging evidence also indicates that the gut microbiota closely regulates intestinal immune homeostasis. Intestinal γδ T cells actively interact with and respond to the gut microbiota, adjusting their functions accordingly.^184,343,470–472^ For instance, the gut microbiota produces short-chain fatty acids (SCFAs), which can suppress IL-17 production by intestinal γδ T cells in patients with IBDs.^473^ Conversely, genetic mutations can increase susceptibility to IBDs by disrupting the regulation of immune responses to pathogenic stimuli.^474–476^ For instance, mutations in genes such as *NOD2*, a cytosolic bacterial sensor, have been identified as high-risk factors for Crohn’s disease.^477–480^ When these genes are mutated, the recognition of gut microbiota by intestinal intraepithelial lymphocytes (IELs), including γδ T cells, becomes dysregulated, leading to the inflammatory pathologies observed in IBDs.^481–483^

In conclusion, intestinal γδ T cells synergistically collaborate with the local immune microenvironment and epithelial cells to uphold symbiosis with the gut microbiota and mount immune responses against invading pathogens. Disruption of this intricate collaboration can lead to IBDs, other intestinal disorders, and even cancers. The recent application of scRNA-seq technology on clinical samples of IBDs has shed light on the complex interaction network among various immune cell subsets at the site of inflammation.^484–488^ This advancement aids in further comprehending the interplay between γδ T cells, the microbiota, and the pathogenesis of IBDs. Additionally, identifying the elusive antigens recognized by γδTCRs in the gut also holds promise for discovering novel therapeutic targets.^184^

#### γδ T cells in multiple sclerosis (MS)

Multiple sclerosis (MS) is a chronic autoimmune disease that affects the central nervous system (CNS), characterized by inflammation, demyelination, and damage to nerve fibers. Its cause involves a combination of genetic and environmental factors.^489–492^ Experimental autoimmune encephalomyelitis (EAE) is an animal model used to study MS, where immunization with myelin antigens induces an autoimmune response against the CNS.^493^ In both MS and EAE, the immune system mistakenly attacks the protective myelin sheath, leading to inflammation and disruption of nerve signals. This results in diverse neurological symptoms, including muscle weakness, sensory disturbances, coordination problems, and cognitive impairments. Early Studies have demonstrated the enrichment and functional characteristics of γδ T cells in the MS and EAE lesions, as well as in the cerebrospinal fluid and peripheral blood of both patients and animal models.^494–499^ Notably, the role of γδ T cells in MS and EAE is controversial, as there is evidence supporting both their protective^326,500^ and pathogenic functions.^501–503^ For instance, γδ T cells regulate the production of IFNγ by T cells infiltrating the CNS and the absence of γδ T cells in TCRδ^−/−^ mice resulted in a more severe course of EAE, like what is observed in mice deficient in IFNγ. This suggests that γδ T cells are important regulators of CNS inflammation and necessary for adequate production of IFNγ in the CNS, which is crucial for the recovery from EAE.^326^ Conversely, conflicting evidence indicated a pathogenic role of γδ T cells instead in CNS inflammation and autoimmunity.^68,504–507^ For instance, IL-1 and IL-23 promote the differentiation of γδT17 cells, which in turn amplifies Th17 responses and contributes to the development of EAE autoimmunity.^62^ Furthermore, it has been shown that IL-23-activated γδ T cells can suppress Foxp3^+^Treg cells, thereby inhibiting the Treg cell-mediated suppression of effector T cell Th17 responses. This disruption in Treg cell function leads to enhanced pathology in EAE.^68^

Recent research advancements have unveiled the regulatory role of γδ T cells in the meninges, the protective membranes surrounding the brain, in modulating brain functions. Under normal conditions, meningeal T cells that produce IFN-γ are involved in the regulation of social behavior,^508^ while meningeal-resident γδT17 cells play a role in modulating anxiety-like behavior,^509^ synaptic plasticity, and short-term memory^510^. Under pathological conditions, γδT17 plays pivotal role in ischemic brain injury^65^ and EAE model,^511^ migrating from the intestine to the meninges after injury and participating in the regulation of aberrant brain functions.^471^ Emerging evidence suggests that changes in the composition and structure of the gut microbiota can have a significant impact on the development and functioning of the host immune system, potentially leading to inflammation in the CNS. One compelling example comes from a mouse model where it was demonstrated that *Lactobacillus acidipiscis*-induced γδ Treg cells can mitigate experimental EAE by suppressing the development of encephalomyelitic Th1 and Th17 cells.^512^ Furthermore, recent research has shown that psychosocial stress can lead to a reduction in *Lactobacillus johnsonii* within the gut microbiota. This reduction, in turn, promotes the differentiation of intestinal γδ T cells into γδT17 cells and their accumulation in the colon. Subsequently, these γδT17 cells migrate to the meninges, establishing a gut-brain axis that mediates the observed depressive behavior.^90,513^ Therefore, a deeper understanding of the regulatory roles played by the gut microbiota could potentially facilitate the development of precise intervention strategies aimed at reconstituting or modifying the microbiota in the treatment of MS.

The clinical implications of γδ T cells in MS are currently being investigated, with their presence and activation at the lesion sites and in peripheral blood suggesting their potential as biomarkers for monitoring disease progression. Furthermore, γδ T cells have been associated with specific clinical features of MS, such as cognitive impairment and disability progression. Advancing our understanding of the role of γδ T cells in MS may facilitate the development of targeted therapeutic strategies.

#### γδ T cells in diabetes

Diabetes is intricately associated with autoimmune diseases, particularly within the realm of type 1 diabetes (T1D). In this specific subtype, the immune system mounts an attack on and ultimately annihilates the insulin-producing pancreatic β cells.^514^ Because insulin is an essential hormone responsible for regulating blood glucose, T1D leads to a shortage of insulin production, which in turn leads to elevated blood glucose levels. The immune response seen in T1D predominantly involves conventional T cells, namely the CD4^+^ helper and CD8^+^ cytotoxic T cells.^515^

Conversely, γδ T cells bridge innate and adaptive immunity by secreting cytokines or acting as antigen-presenting cells. They are thought to regulate T and B cell responses in T1D. Notably, γδ TCRs possess a broader antigen recognition repertoire than αβ TCRs and are MHC-unrestricted, enabling them to directly recognize T1D-associated antigens.

Early studies indicated deficient αβ but not γδ TCR thymocyte development in the non-obese diabetic (NOD) mouse model, suggesting distinct regulation of these T cell populations in diabetic milieu.^516,517^ Moreover, thymic αβ/γδ-lineage decision skews towards αβ in diabetes-prone NOD mice, revealing thymic selection anomalies.^518^

Notably, γδ T cells in NOD mice recognize processed insulin like αβ counterparts.^519^ Aerosolized insulin induces regulatory CD8^+^γδ T cells in NOD mice, preventing diabetes onset.^49^ Furthermore, reduced CD8^+^ and CD8^−^γδ T cells were observed in prediabetic individuals.^520^ A longitudinal study established the temporal association between γδ T cell percentage and the onset of T1D. Cumulatively, these studies provide further endorsement for the regulatory and hence protective role played by γδ T cells in T1D.^521–523^

However, the introduction of TCRδ-deficiency onto the NOD mouse background shields them from T1D, thereby hinting at the pathogenic role of γδ T cells.^524^ Moreover, a recent study illuminated the dualistic, both protective and pathogenic, role that γδ T cells enact in T1D contingent upon their functional subsets.^525^ As such, the precise role of γδ T cells in diabetes remains to be clarified and may pivot on specific contextual factors. Additionally, the involvement of γδ T cells in type 2 diabetes (T2D)^526,527^ remains relatively unexplored, with a scarcity of available literature to facilitate in-depth discussions. This situation underscores the need for additional investigations to shed light on this aspect.

### γδ T cells in cancers

#### Elements of tumor microenvironment (TME) attenuate γδ T cell functions

It has been well acknowledged that the TME is detrimental to the T cell-mediated tumor immunosurveillance.^2,3,528^ The functional polarization of γδ T cells by various TME elements results in their pleiotropic effector functions in cancers (Fig. 3a).^529^ Here, we briefly list some well-known TME features contributing to the modulation of T cell immunity.

##### Epigenetic regulation

Recently, it has been discovered that epigenetic and transcriptional regulations have an impact on the functional differentiation of γδ T cells.^171,317,530^ In our recent review, we elaborated on epigenetic modulators in the TME that can initiate a functional shift in infiltrated T cells.^531^ For example, lactate, alpha KG, and acetyl-coa can regulate various histone modifications, thus affecting transcription factor(s) binding. This can result in either the “silencing” or “activation” of gene expression in γδ T cells, similar but through different regulatory transcription factors or cytokines when compared with αβ T cells.^317,532^ However, most of the previous studies were conducted using mice models, and there are significant differences in the functional regulation and differentiation of γδ T cells in mice and humans. Therefore, more studies using human samples are needed.

##### Hypoxia

Similar to αβ T cells, γδ T cells primarily rely on glycolysis rather than mitochondrial respiration to carry out their effector responses. This metabolic shift occurs as naïve cells differentiate into effector cells. However, TME poses challenges for both glycolysis and oxygen availability, severely impairing the anti-tumor effector function, survival capacity, and proliferation/differentiation potential of naïve γδ T cells. In a brain tumor model, a hypoxic TME was found to impair the effector function of γδ T cells, while αβ T cells were unaffected.^533^ Conversely, Siegers et al. reported enhanced cytotoxicity but reduced proliferation of γδ T cells under hypoxic conditions in vitro.^534^ These seemingly contradictory observations can be attributed to the heterogeneity of the TME across various cancer types. Therefore, further research is crucial to unravel the complex interactions and design therapeutic regimens that are tailored to the specific TME characteristics of individual cancer patients.

##### Oxidative stress

Dysregulated Reactive oxygen species (ROS) in TME have long been considered to negatively impact T cell-mediated anti-tumor immunity.^535^ Nonetheless, tumor-associated neutrophils-derived ROS could restrain the pro-tumoral effect of γδT17 cells.^536^ Further understanding of the roles of ROS in tuning γδ T cell functions might benefit their clinical application.^537^

##### Exosomes

Tumor-derived exosomes (TDE) play an important role in the development of tumor immune escape.^538^ The TDE has been shown to regulate the pro- or anti-tumor responses of γδ T cells.^539^ Ni et al. showed that cancer cell-secreted exosomes upregulated the immunosuppressive CD73^+^Vδ1^+^ TILs (Treg) population via exosome-embedded lncRNA SNHG16 in breast tumors.^313^ Moreover, a study showed that tumor-derived exosomes could induce MDSC-directed γδ T exhaustion.^539^ Interestingly, a study published by Tu’s group showed that, exosomes derived from Vδ2 T cells exhibit strong anti-tumor potentiality as well.^540^ However, how to utilize exosomes (tumor- or immune cell derived) to further potentiate clinical efficacy of adoptive transferred allogeneic Vδ2 T cells remains to be fully addressed.

##### Treg

Tumor-infiltrated CD4^+^Treg has been shown to inhibit the anti-tumor immunity of γδ T cells in HCC through the secretion of TGFβ and IL-10.^541^ Additionally, tumor-derived TGFβ can induce the differentiation of immunosuppressive CD39^+^ γδTreg cells in colorectal cancer (CRC).^314^ Circulating neutrophils^542–544^ and MDSCs^545^ in the TME can also restrain the anti-tumor response of γδ T cells.^546^ Therefore, fully deciphering the immune landscape of TME and elaborating the interactions between immunosuppressive cell populations and γδ T cells ensure further understanding of γδ T cell functions in TME.

##### Checkpoint molecules

In the context of TME, another important feature is the elevated expression of checkpoint molecules, which are involved in dampening the effector capabilities of tumor-infiltrating γδ T cells. The principal co-inhibitory molecules expressed in T cells predominantly encompass PD1 (Programmed Cell Death Protein 1), LAG3 (Lymphocyte-Activation Gene 3), CTLA4 (Cytotoxic T-Lymphocyte Associated Protein 4), TIM3 (or HAVCR2; T cell immunoglobulin and mucin-domain containing-3), TIGIT (T cell immunoreceptor with immunoglobulin and ITIM domains), BTLA (B and T lymphocyte attenuator), B7-H3 (CD276), and others. These pivotal checkpoint molecules have been documented to assume crucial roles in curtailing T cell cytotoxic functions.^547–549^ In the case of γδ T cells, these checkpoint molecules also govern cellular effector function. For instance, PD1 and TIM3 can differentially modulate the anti-tumor activity of specific subsets of murine γδ T cells, namely Vγ6^+^ and Vγ4^+^ cells, which produce IL-17A.^550^ In human, the exhaustion of intratumoral γδ T cells correlates with the expression of various immune checkpoints such as PD1, TIGIT, TIM3, CTLA4, and CD39.^551,552^ As for BTLA, it negatively regulates human Vδ2 T cell proliferation^553^ and curbs γδ T cell numbers and sustains normal frequencies of γδ T cell subsets. As a results, it maintains the equilibrium of γδ T cell populations and controls inflammatory responses in mice.^554^

In the case of B7-H3, an immunoregulatory protein belonging to the B7 family, it is expressed on T cells. B7-H3 can suppress the cytotoxicity of human Vδ2 T cells by downregulating the expressions of IFN-γ, perforin, and granzyme B.^555^ Intriguingly, TIM3 not only fulfills roles in modulating the function of γδ T cells in tumors but also reduces inflammatory reactions of γδ T cells. Consequently, this leads to a reduced susceptibility to malaria infection and minimized malaria symptoms in children.^556^

Furthermore, according to our work, we propose that LAG3 holds promise as a target checkpoint in solid tumor, particularly in HCC. This assumption is grounded in our published data, which indicate that LAG3, rather than other molecules such as TIM3 and PD1, is notably upregulated in HCC-infiltrating γδ T cells. Additionally, a similar phenotype can be induced through glutamine restriction.^243^ Nonetheless, given the intricate nature of the TME, multiple checkpoint molecules, as opposed to a single entity, contribute to impairing the effector function of γδ T cells. Thus, we propose that a prospective strategy for tumor immunotherapy shall involve the simultaneous blockade of multiple checkpoint targets and the adoptive transfer of γδ T cells derived from healthy donors.

#### γδ T cells in hematological cancers

Hematologic cancer, also known as hematological malignancy or blood cancer, encompasses a diverse group of neoplastic disorders affecting the blood, bone marrow, and lymphatic system. This category includes leukemia, lymphoma, and multiple myeloma. These malignancies originate from aberrant growth and differentiation of blood cells, leading to perturbations in normal hematopoiesis and hematologic function.^557–559^ Hematologic cancer has multifaceted causes, encompassing genetic^560–564^, environmental,^565,566^ and lifestyle factors,^567^ etc. Viral infections, for example, have been associated with a higher risk of specific hematologic cancers.^243,568,569^ Additionally, autoimmune diseases, immunodeficiency disorders, and chronic inflammatory conditions can heighten the susceptibility to hematologic cancer.^570–574^ Together, these factors contribute to the development of hematologic malignancies. Although progress has been made in the long-term survival of the patients,^575^ the inherent complexity and heterogeneity of hematologic cancer makes it difficult to develop universal treatment strategies. In the context of hematological malignancies, observations have been made regarding functional deficiencies of γδ T cells.^576^ Studies have demonstrated the dual roles of γδ T cells, exhibiting both anti-tumor^577–581^ and pro-tumor^582^ functions. However, it is important to note that these functional outcomes are highly dependent on the context and functional characteristics of the γδ T cell subsets involved.

Early studies focused on stimulating in vivo or ex vivo expansion of γδ T cells of patients.^583^ Nevertheless, one of the main drawbacks of using autologous γδ T cells in cancer treatment is the compromised function of γδ T cells in cancer patients, not to mention the systemic side effects of the drugs used to stimulate γδ T cell proliferation, such as zoledronate,^584,585^ which ultimately leads to limited clinical benefits.^54,586–589^ Furthermore, γδ T cell recognition of malignant cells does not depend on MHC presentation, meaning they would theoretically not recognize the recipient as “non-self” and mount immune attacks.^9,14^ This unique property provides an advantage in utilizing allogeneic γδ T cells for cancer treatment and bypassing the graft-versus-host effects associated with MHC-mismatched αβ T cells.^590,591^ Additionally, early clinical observations indicated that increased γδ T cell levels (particularly the Vδ1^+^ subset) predicted long-term disease-free survival in acute leukemia patients following Allogeneic stem cell transplantation (ASCT).^592–595^ These findings prompted successful attempts to utilize haploidentical stem cell transplantation (HSCT) in treating pediatric patients with acute leukemia (NCT01810120). The approach involved depleting αβ T and B cells using antibodies while preserving only the mature immune-competent γδ T cell and NK cell populations. In this study, during the early post-transplantation period, the Vδ1^+^ and Vδ2^+^ subsets were predominantly composed of central-memory cells. Interestingly, the differentiation status persisted in the Vδ2^+^ subtype even six months after transplantation, while the Vδ1^+^ subtype exhibited a drastic decrease in central-memory cells but an increase in terminally differentiated cells by the sixth month. Furthermore, a significant increase in the percentage of the Vδ1^+^ subset, accompanied by a decrease in the Vδ2^+^ subset, was demonstrated, suggesting diverse functional roles between these two subsets.^596^ A follow-up study further demonstrated a 5-year probability of chronic graft-versus-host disease (GVHD)-free, relapse-free survival (GRFS) at 71%, comparable to that of HLA-matched donor HSCT recipients, indicating long-term benefits of allogeneic γδ T cells graft.^597^ A similar clinical trial was conducted in adults with hematological malignancies.^598^ In all cases, early reconstitution of γδ T cells was observed after HSCT, along with prognostic benefits such as reduced risk of infections and improved event-free survival, emphasizing their functional roles following allogeneic HSCT for leukemia.^595,599–601^ Currently, multiple clinical trials are underway to directly transfer allogeneic γδ T cells to exert a graft-versus-leukemia (GVL) effect, either Vδ1^+^ or Vδ2^+^ subset (phase I clinical trial NCT03790072, NCT03533816), to patients with hematologic cancer.^602,603^ Additionally, an intriguing and promising prospect of applying allogeneic γδ T cell immunotherapy is the treatment of patients with malignant γδ T cell transformation, such as hepatosplenic γδ T cell lymphoma (HSGDTL),^604–607^ primary cutaneous γδ T cell lymphoma (PCGDTL),^608,609^ and acute lymphoblastic leukemia (γδ T-ALL).^8,610–612^

Other than HSCT, another promising immune cell-based immunotherapy to treat hematological cancers is CAR-T cell Therapy.^23,613^ CAR T therapy is a groundbreaking evolution in cell-based immunotherapy pioneered by Dr. Carl June in treating hematological malignancies, such as chronic and acute leukemia like acute lymphoblastic leukemia (ALL) and chronic lymphocytic leukemia (CLL) etc. CD19-directed CAR-T cell therapy has demonstrated remarkable efficacy, with an overall remission rate (OR) as high as 81% within 3 months in the treatment of B-Cell Lymphoblastic Leukemia.^614,615^ This efficacy is attributed to the efficient recognition and binding of CD19 CAR-T cells to CD19-expressing malignant B cells, leading to their targeted destruction.^21,22,616–619^ However, the application of CAR-T therapy is hindered by systemic side effects, such as cytokine storm syndrome, a systemic immune dysregulation that can result in multiorgan failure if left untreated.^415,620^ Additionally, neurotoxicity has been reported in over 60% of patients receiving CAR-T therapy.^621–623^ This neurotoxicity is mainly caused by the ability of CAR-T cells to trigger cytokine release syndrome (CRS) and migrate to the central nervous system (CNS), targeting and compromising the integrity of the blood brain barrier (BBB),^624^ which enable them to further interact with neuronal cells and activate brain-resident immune cells, leading to an inflammatory response and subsequent neurotoxicity.

Recently, CAR-transduced γδ T cell-based immunotherapy has garnered attention due to several unique advantages. Firstly, γδ T cells exhibit a wide recognition spectrum for tumor-associated antigens, encompassing stress-induced ligands, phosphoantigens, and non-peptide antigens. This expanded recognition capacity positions γδ T cells favorably compared to αβ T cells in the realm of cancer immunotherapy. Additionally, γδ T cells can exert tumor-toxicity through direct engagement with cancer cells or by activating other immune cells. Furthermore, their MHC-unrestricted recognition of malignant cells reduces the likelihood of graft-versus-host disease (GVHD)^599^ and enables them to overcome immune evasion strategies employed by cancers, such as downregulated MHC molecule expression. Although CAR γδ T cell immunotherapy is still in its early stages of development, several promising studies have emerged. For instance, studies demonstrated reduced tumor burden in mice using CD19-specific CAR-T cells in leukemia model.^625,626^ Furthermore, recent research by Pablo et al. showcased the efficacy of allogeneic CD123 CAR-Delta One T (DOT, Vδ1^+^) cells, which target the interleukin-3α chain receptor (CD123) expressed on acute myeloid leukemia (AML) blasts, in the treatment of AML in mice.^627^ Additionally, allogeneic CD20-targeted CAR Vδ1^+^ γδ T cells, specifically designed to target the B-cell-restricted CD20 antigen, exhibited anti-tumor activity in a B-cell lymphoma mouse model. A phase I trial is currently underway to evaluate the efficacy of these CAR T cells in patients with relapsed/refractory B-cell malignancies (NCT04735471).^628^

To conclude, hematologic cancers encompass a wide array of neoplastic disorders affecting the blood, bone marrow, and lymphatic system. Their complex etiology involves genetic, environmental, and lifestyle factors, with viral infections and immune-related conditions contributing to susceptibility. Advances have improved patient survival, yet the intricate nature of these malignancies hinders universal treatment strategies. Observations on γδ T cells reveal their dual roles in cancer, but context-specific functions underscore the need for deeper understanding. Allogeneic γδ T cells show promise, as seen in HSCT trials, offering advantages over conventional approaches. Furthermore, CAR-γδ T cell therapy emerges with expanded recognition and potential benefits. Despite progress, in-depth investigations, including single-cell transcriptome analysis,^629–631^ remain crucial to fully exploit γδ T cells’ potential and advance targeted therapies for hematologic malignancies.

#### γδ T cells in solid tumors

γδ T cells hold great potential as a novel immunotherapeutic approach for not only hematological cancers but also solid tumors. While they increasingly exhibit remarkable potential in the immunotherapy of hematological malignancies as discussed above, γδ T cells also represent the future in solid tumor immunotherapy. It has been recognized that γδ T cells possess a remarkable ability to identify stress-induced antigens on tumor cells, even in scenarios involving low mutational burdens or MHC defects,^256^ rendering them a valuable approach for solid tumor therapy. Tumor cells frequently downregulate HLA class I (MHC-I) to evade the immune response, which impedes the conventional activation of CD8^+^ T cells. This distinctive trait of γδ T cells becomes particularly advantageous in augmenting the scope of existing T cell-based immunotherapies. For instance, T cell receptor-engineered T cell (TCR-T) therapy primarily focuses on antigens presented through HLA class I molecules. However, in cancers where HLA class I is deficient, TCR-T cells might encounter challenges in recognizing antigens. Integrating the autonomous antigen recognition capability of γδ T cells, independent of HLA class I, into the development of the γδ TCR-T will enable evasion of immune evasion mechanisms caused by diminished HLA expression in cancer cells. Additionally, the identification of individuals with tumors lacking HLA class I would enable the personalized utilization of CAR- or TCR-γδ T cells, ensuring treatment alignment with the tumor’s immune characteristics.

The unique MHC-independent recognition also offers the advantage of reduced immune rejection, rendering allogeneic adoptive γδ T cell transfer a safer therapeutic approach.^11,12,587^ Additionally, γδ T cells exhibit efficient APC capabilities, effectively activating other immune cells to mediate tumor clearance.^56,217,632^ By functioning both as tumor-specific effectors and potent APCs, γδ T cells hold significant promise in the field of immunotherapy for both hematological cancers and solid tumors.

Currently, there are two main categories of γδ T cell-based therapies which include γδ T cell engagers and adoptive γδ T cell transfer^9^. The cell engagers strategy primarily involves the use of mono- or bispecific antibodies to connect γδ T cells with their targets, leading to highly specific tumor lysis. For example, the use of an agonistic BTN3A1 antibody, which binds to BTN3A1^+^ cancer cells, triggers phosphoantigen-like Vγ9Vδ2 T activation and tumor recognition^82,633^ A phase I/IIA clinical trial of this strategy is currently underway (NCT04243499). Additionally, bispecific antibodies designed to bind both the γδ TCR (mainly Vγ9) and cancer-specific target molecules, such as HER2,^634^ CD123,^635^ EGFR,^636^ CD40,^637^ and CD1d,^638^ are also being developed. Interestingly, bispecific antibodies targeting both cancer phosphoantigen-sensing Vγ9 TCR and CD3 binding domains have demonstrated enhanced effectiveness in αβ T cell-mediated cancer-killing, indicating that Vγ9 TCRs act as a “cancer detector” and recruit αβ T cells to the tumor microenvironment.^639^ Furthermore, in addition to γδ T cells, novel bispecific engager strategies^640^ might also simultaneously recruit other innate-like effector cells.

On the other hand, adoptive cell therapy is further divided into two types, naturally expanded or genetically modified γδ T cell strategy.^641^ Early studies showed limited success in using in vivo synthetic phosphoantigen-stimulated or cancer patient-derived ex vivo expanded autologous γδ T cell transfer,^14,583,587^ mainly due to the impaired immunity of patients. Therefore, the focus has shifted to allogeneic γδ T cell transfer. Early attempts were made in hematological malignancies using allogeneic stem cell transplantation depleted for αβT cells^627,628^or haploidentical γδ T cells,^603^ which showed reasonably high objective response (OR) rate with limited side effects. Therefore, our research team pioneered the first clinical allogeneic Vδ2 T cells transfer on 132 patients with various terminal solid tumors. The observed clinical benefits through a total of 414 cell infusions established a proof-of-concept for the application of allogeneic Vδ2 T cells in solid tumor treatment.^11,12^ Additionally, clinical applications of allogeneic Vδ1^+^ T (DOT) cells showed promising results in hematological malignancies.^642,643^ Unlike the Vδ2^+^ subtype, Vδ1^+^ T cells seem to resist AICD,^248,279^ which might provide persistent protection. A previous study also showed the superior tumor cytotoxicity of Vδ1^+^ over Vδ2^+^ in a mouse xenograft tumor model.^644^ Therefore, further functional comparisons between these two subtypes could help gain insights into their respective clinical benefits.

Along with the natural expansion strategy, CARs-transduced γδ T cells have been developed with well-known targets on solid cancers, such as GPC3^27^ and NKG2D ligand.^645^ However, it has been found that the cancer cell cytotoxicity of CAR- Vγ9Vδ2 T gradually diminishes, raising concerns about Vγ9Vδ2 clinical persistence.^646^ Another type of CAR modification is to fuse γδ TCR with αβ T cells, namely γδ TCR-engineered T cells.^271,647,648^ This strategy was deployed in various cancer models,^649^ and phase I clinical trials are ongoing. Table 1 lists ongoing or completed clinical trials on allogeneic γδ T cell-based cancer therapy. Although promising preclinical results were demonstrated, further evidence is needed to establish both the safety and efficacy profile of these genetically modified γδ T cell strategies. Finally, adoptive γδ T cell transfer could be applied in combination with immune checkpoint inhibitors (ICIs: PD-1, CTLA4, LAG3, etc.) to maximize cytotoxic potency and avoid exhaustion.^247,650^ Nonetheless, both γδ T cell engager and adoptive γδ T cell transfer strategies require further clinical validations.

#### Allogeneic γδ T cells: off-the-shelf medicine for tumor immunotherapy

**Fig. 7:**
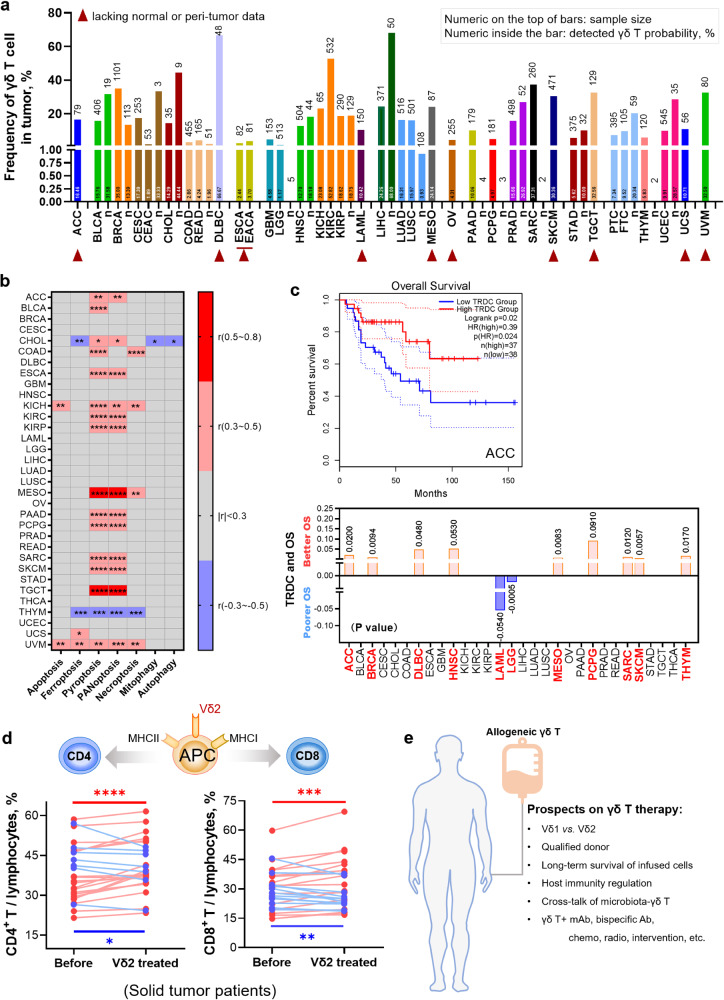
The correlation between tumor-infiltrating γδ T cells and cancer pathogenesis, along with the clinical potential of allogeneic γδ T cells in tumor immunotherapy. According to our recent TCGA-based bioinformatics analysis (**a**–**c**) and revisiting our previous clinical trial data records (**d**),^11,12^ allogeneic γδ T cell indicates the future of tumor immunotherapy. **a** The abundance of γδ T cells in normal and tumor tissue depends on the type of organ (‘n’ represents normal). **b** Correlation analysis between *TRDC* and programmed cell death (PCD) gene sets show variability across cancers. Nevertheless, pyroptosis and PANoptosis of γδ T cells appear to be more correlated with cancers than other PCD pathways. PCD gene set lists are provided in the supplemental file. The correlation coefficient is represented by “r”. **c** The infiltrated γδ T cells (*TRDC*) can serve as an indicator for the overall survival (OS) in a very small fraction of cancer types (11/33), and only correlate with better OS in 9/33 cancers. **d** Our clinical trials have shown that allogeneic Vδ2 T therapy significantly increases the percentages of CD4^+^ and CD8^+^ T cells in certain patients with solid tumors (represented by red lines in the graph, each line representing data from an individual patient). **e** A sketch graph of allogeneic γδ T cell-based cancer immunotherapy is presented, along with potential challenges and γδ T^plus^ strategy (γδ T + mAb, bispecific Ab, chemo, radio, intervention, etc.) in clinical application. *p < 0.05; **p < 0.01; ***p < 0.001; ****p < 0.0001. ACC Adrenocortical carcinoma, BLCA Bladder Urothelial Carcinoma, BRCA Breast invasive carcinoma, CESC Cervical squamous cell carcinoma, CHOL Cholangio carcinoma, COAD Colon adenocarcinoma, DLBC Lymphoid Neoplasm Diffuse Large B-cell Lymphoma, ESCA Esophageal squamous cell carcinoma, GBM Glioblastoma multiforme, HNSC Head and neck squamous cell carcinoma, KICH Kidney chromophobe, KIRC Kidney renal clear cell carcinoma, KIRP Kidney renal papillary cell carcinoma, LAML Acute myeloid leukemia, LGG Brain lower grade glioma, LIHC Liver hepatocellular carcinoma, LUAD Lung adenocarcinoma, LUSC Lung squamous cell carcinoma, MESO Mesothelioma, OV Ovarian serous cystadenocarcinoma, PAAD Pancreatic adenocarcinoma, PCPG Pheochromocytoma and paraganglioma, PRAD Prostate adenocarcinoma, READ Rectum adenocarcinoma, SARC Sarcoma, SKCM Skin cutaneous melanoma, STAD Stomach adenocarcinoma, TGCT Testicular germ cell tumors, THCA Thyroid carcinoma, THYM Thymoma, UCEC Uterine corpus endometrial carcinoma, UCS Uterine carcinosarcoma, UVM Uveal melanoma

For cancer patients, the impaired function of γδ T cells and the difficulty in expanding circulating Vδ2 T cells for autologous immune cell therapy have been observed in our preclinical studies. Additionally, the tumor microenvironment not only suppresses the function of γδ T cells but also reduces their cell number with programmed cell death, including AICD, playing a crucial role in the reduction of infiltrated γδ T cells, according to our published work.^243^ However, it is difficult to conclude the abundance of TME-infiltrated γδ T cells between normal and cancer tissue using TCGA-based data analysis (Fig. 7a). Notably, TCGA analysis suggested a significant correlation between TRDC and the gene sets of pyroptosis and PANoptosis in most carcinomas (Fig. 7b), indicating programmed cell death of γδ T cells in the TME. Nevertheless, this analysis cannot determine which subset of γδ T cells is more tolerant in the TME.

Additionally, although infiltrated-γδ T cells have been identified as the most favorable indicator for good prognosis in 25 types of cancers,^86^ over-mining of TCGA data may lead to biased and controversial conclusions.^339^ Therefore, we conducted a straightforward assay to investigate the relationship between TRDC and the overall survival (OS) of pan-cancer patients based on the TCGA database. We found that, among 33 types of cancers, *TRDC* is correlated with good prognosis (better OS) of only 9 types of cancers, and two types has worse OS (Fig. 7c). However, TCGA database-based bioinformatics can still provide some valuable directional cues for understanding γδ T cell immunity in the context of the TME. We anticipate that the latest scRNA-seq technology will help uncover the comprehensive signatures of γδ T cells in both healthy individuals and tumor patients.

Even though γδ T cells are numerically reduced and functionally impaired in the TME, intra-tumoral infiltration is positively correlated with good prognosis among most cancers. γδ T cells, particularly allogeneic γδ T cells-based immunotherapy, represent a new alternative treatment for cancer patients. For example, adoptive transfer of in vitro-expanded allogeneic Vδ2 T cells not only controls tumor progression and achieves remission in some patients with solid tumors,^11,12^ but may also serve as APCs to elevate the percentages of CD4^+^ and CD8^+^ T cells (red dots/lines in Fig. 7d) according to our study. Meanwhile, one of the major functions of Vδ2 T cells is to secrete IFNγ, which plays a crucial role in regulating αβT cells as well. Preliminary evaluation based on our clinical data showed that most of those patients who had elevated CD4^+^ and/or CD8^+^ T cells had an extended overall survival, suggesting that the regulation of αβT cell percentage might be a response indicator for allogeneic Vδ2 T cell therapy. Notably, this endorses that although γδ T cells recognize and kill target cells in a MHC-independent manner, MHCs of γδ T cells functionally play crucial roles in regulating other types of immune cells, such as αβT cells. Remarkably, that MHC-independent recognition pattern highlighted the unique advantage of no acute graft-versus-host disease (GVHD) of γδ T cells. Together, we believe that the unique advantages of γδ T cell-based cancer immunotherapy cannot be replaced by other types of immunotherapies, and represents a key future for tumor immunotherapy.

Additionally, it is interesting to highlight the similarity between γδ T cells and NK cells in their recognition and elimination of stressed or transformed cells, encompassing cancer cells and pathogen-infected cells, through an array of activating and inhibitory receptors. Notably, NK cells have been comprehensively reviewed recently.^651,652^ Overall, both γδ T and NK cells exhibit a broader spectrum of tumor cell recognition compared to conventional αβ T cells. The main difference between the two cell types refers to the expression of the γδ TCR which is missing on the CD3-negative NK cells. As a consequence, both cell types identify stress-induced ligands via activating and inhibitory NK receptors, but only γδ T cells recognize tumor cells on the basis of enhanced phosphoantigen production. While γδ T cells act independently of MHC restriction, the activation of NK cells is intimately regulated by receptors which sense dysregulated HLA class I expression and/or stress-induced ligands on cancer cells. The use of either cell type mitigates risks of alloreactivity and graft-versus-host disease (GvHD).^601,651,653^ Diverse innate cytotoxicity receptors on their cell surfaces equip them to detect a wide range of cancer antigens. These qualities support the development of allogeneic cell therapies involving γδ T cells or NK cells, with applicability to diverse malignancies.^652^ At present, both γδ T cells and NK cells are extensively explored in CAR-based therapies.^26,279,651,654^ However, variations may exist in their antigen recognition. Particularly noteworthy is that γδ T cells, but not NK cells, serve as professional antigen-presenting cells,^56,354^ exerting pivotal roles in regulating immune responses in cancers. Despite shared features between these two immune cell types, distinctions in antigen recognition mechanisms and immune attributes might influence the precision of CAR-NK and CAR-γδT therapies when targeting malignancies. The choice of CAR antigens and the characteristics of the tumor microenvironment can impact treatment efficacy. Nonetheless, in-depth research is indispensable to fully comprehend the potential of γδ T and NK cells in the context of targeting malignancies.

Finally, in order to provide a comprehensive overview of the current advancements in allogeneic γδ T cell-based immunotherapy for cancer, we have compiled and presented a summary of registered clinical trials available on the clinicaltrials.gov website, as shown in Table 2.

**Table 2:** Summary of allogeneic γδ T cell-based cancer therapy in clinical trials

Tumor type	NCT	Status	Therapy	Recruit number	Start date	Location	Supplementary
Breast	NCT 03183206	Completed	Procedure: Cryosurgery or IRE surgery. Biological: Allogeneic γδ T cell. Other: Allogeneic γδ T cells/A Cryosurgery or IRE.	100	June, 2017	Guangdong, China	~1.5 × 108 γδ T cells every 2 wk. Cell culture: RPMI-1640, IL-2, IL15, zoledronate, vitamin C
Liver	NCT 03183219			30	June, 2017	Guangdong, China	
Lung	NCT 03183232			30	June, 2017	Guangdong, China	
Pancreatic	NCT 03180437			62	June, 2017	Guangdong, China	
Malignant Solid Tumor	NCT 04765462	Recruiting	Allogeneic γδ T cells	60	March, 2021	Beijing, China	In the process of a dose escalation trial
AML; ALL; Myelodysplastic Syndromes;	NCT 04764513	Recruiting	Ex-vivo expanded γδ T cell infusion	20	Sept, 2021	Beijing, China	
AML	NCT 04008381	Unknown	Ex-vivo Expanded γδ T Lymphocytes	38	Sept, 2019	Wuhan, China	
AML	NCT 05358808	Recruiting	Ex-Vivo Expanded Allogeneic γδ T-lymphocytes (TCB-008)	148	Aug, 2022	UK	7 × 10^7 or 7 × 10^8 cells
AML	NCT 03790072	Completed	Ex-vivo Expanded γδ T-lymphocytes (OmnImmune®)	10	Nov, 2018	Czechia	Dose escalation
Glioblastoma	NCT 05664243	Not yet recruiting	Autologous/ Allogeneic genetically modified γδ T cells	120	Jan 2023	Alabama, US	/
AML	NCT 05015426	Recruiting	γδ T-Cell Infusion	32	Aug, 2021	Florida, US	Dose escalation
Neuroblastoma; Refractory/Relapsed Neuroblastoma	NCT 05400603	Not yet recruiting	Ex Vivo Expanded Allogeneic γδ T Cells in Combination with chemotherapy	24	Nov, 2022	Georgia, US	Dose escalation
Non-Hodgkin’s Lymphoma (NHL); Peripheral T Cell Lymphoma (PTCL)	NCT 04696705	Recruiting	Ex-vivo expanded allogeneic γδT cells	10	Dec, 2020	Tianjin, China	Dose escalation
HCC	NCT 04518774	Unknown	Ex-vivo expanded allogeneic γδT cells	8	Aug, 2020	Beijing, China	
AML, CML, ALL Myelodysplastic Syndromes	NCT 03533816	100%CR in AML declaimed	Expanded/Activated γδ T-cell Infusion	38	Jan, 2020	Kansas, US	1, 3, or 10 × 10^6 cells/kg. Cell expansion: CliniMACS-Prodigy
Cancer;Malignancy; Refractory/Relapsed Cancer	NCT 05302037	Not yet recruiting	Allogeneic NKG2DL-targeting CAR-grafted γδ T Cells (CTM-N2D)	9	April 2022	Singapore	Four infusions: 1 × 10^7, 1 × 10^8, 3 × 10^8 or 1 × 10^9 per infusion every 7 days
Colorectal; TNBC; Sarcoma; Prostate; Nasopharyngeal Carcinoma; Gastric	NCT 04107142	Unknown	Biological: Adoptive Cell Transfer of NKG2DL-targetting CAR-grafted γδ T cell	10	Dec, 2019	Malaysia	“3 + 3” dose escalation: 3 × 10^8 - 3 × 10^9
Lymphoma	NCT 04735471	Recruiting	Anti-CD20 Allogeneic CAR- γδ T with chemotherapy	78	March, 2021	US	“3 + 3” Dose Escalation
B-cell Leukemia	NCT 04439721	Unknown	Allogenic γδT Cell infusion agent	5	May, 2020	Jiangsu, China	0.5 × 10^6-8 × 10^7γδT /kg, once
Non-Hodgkin’s Lymphoma	NCT 05554939	Recruiting	Allogenic CD19-CAR-γδT cell with chemotherapy	30	Dec, 2022	Beijing, China	“3 + 3” Dose Escalation
Leukemia Lymphoma	NCT 02656147	Unknown	Allogeneic Anti-CD19-CAR γδT	48	Oct, 2017	Beijing, China	Dose escalation

Note: Conclusion of these study is not available yet except NCT03533816

## Future prospects

Based on our previous clinical observations,^11,12^ it has been found that allogeneic Vδ2 T cell transfer, derived from healthy donors and administered to cancer patients, is safe. However, it has also been observed that only a fraction of patients responded well to the treatment. Therefore, we have summarized a few key challenges that need to be addressed in order to ensure successful allogeneic γδ T cell clinical applications (as depicted in Fig. 7e).

### Qualified donor selection

One challenge is if and how to match donors with recipients to guarantee therapeutic benefits. Due to their MHC-independence, HLA-matching may not be required, but there are as yet scarce data available to judge whether full allogeneic mismatch or haplo-identical transfers are preferable. For clinical application of allogeneic Vδ1^+^ T cells, the matching strategy might be solved by sequencing the γδTCR on donor γδ T cells to selectively expand subclones with strong functional activities,^271,655^ which can recognize and attack the tumor-associated antigens (TAAs) and/or neoantigens of the patients. Interestingly, evidence has shown that human γδTCR displayed cross-reactivity with CMV-infected cells and tumor cells,^656–658^ implying that previous infection history of the donors might partially affect the effectiveness of donor γδ T cells towards cancer patients. For Vδ2 T cells, however, the strategy may focus on examination of the expression level of tumor-derived phosphoantigens, which helps the physician decide what type of cancers or which individual patient is more suitable for this therapy. Notably, during the past few years, our group developed a strategy for examining the immune phenotypes of circulating immune cells based on flow cytometry assay, which can help analyze the function of each cell population. This approach enables us to perform donor-recipient matching. Altogether, further exploration of functional “biomarkers” can help develop personalized and precision treatment regimens to maximize the efficacy of γδ T-based cell therapy.

### Vδ1^+^ vs Vδ2^+^: two branches of tumor immunotherapy

Another challenge is which γδ T subtype is more effective for tumor therapy. Careful evaluations of chemotaxis ability, durability, and tumor-cytotoxicity need to be established for both hematological and solid cancers to compare the clinical benefit and safety of Vδ1 and Vδ2 T cell subsets. For instance, although both subtypes share a suite of chemokine receptors on their surface, CCR5 is restricted to Vδ2 T cells, while CXCR1 is mainly expressed on Vδ1^+^ cells of circulating blood.^188,190^ In addition, tumor-infiltrating Vδ1^+^ cells highly express CXCR3.^308^ These findings suggest different tissue migratory patterns of Vδ1^+^ and Vδ2^+^ subsets when they receive inflammatory signals. Furthermore, dysregulated profiles of chemokine and chemokine receptor expression in γδ T cells can contribute to disease progression.^659^ Notably, adverse factors in the TME discussed above might “manipulate” γδ T cell migration patterns toward a pro-tumorigenic one. Since the chemokine landscape helps determine immune cell chemotactic migration and retention within the TME, which further shapes the pro- or anti-tumor responses in a spatiotemporal manner,^660,661^ a thorough understanding of γδ T cell chemokine receptor profiles and factors orchestrating γδ T cell chemotaxis, especially the tumor trafficking properties of both Vδ1^+^ and Vδ2^+^ subsets, might benefit the advancement of allogeneic γδ T cell-based cancer immunotherapy.

### Clinical efficacy evaluation

Clinical efficacy evaluation in tumor cell therapy mainly involves the applications of common criteria, Response Evaluation Criteria in Solid Tumors (RECIST), including assessments of objective tumor response (tumor size, volume, or radiographic imaging) that is applied to classify responses as complete response, partial response, stable disease, or progressive disease, overall survival (OS), progression-free survival (PFS), quality of life (QoL), adverse events (AEs), and biomarker analysis. These parameters provide insights into treatment response, patient outcomes, safety, and the therapy’s impact on the patient’s well-being. By employing rigorous scientific methodologies, researchers and clinicians can make evidence-based decisions regarding the efficacy of tumor cell therapy in immunotherapy. However, comprehensive approaches are needed to assess the long-term persistence and functionality of γδ T cells in vivo, including their ability to establish durable memory responses and exhibit APC-like properties, which is crucial to understand their roles in shaping overall patient immunity in addition to tumor cell killing. Previously, we used immunophenotypes to assess the immune status of patients before and after allogeneic γδ T cell transfer, which can reveal significant perturbation in their immune profile (as shown in Fig. 7d). Utilizing advanced immunological techniques such as single-cell multi-omics spatiotemporal analyses and robust experimental models, researchers can gain deeper insights into the immunological mechanisms and therapeutic potential of γδ T cells, further paving the way for enhanced patient care and tailored immunotherapeutic strategies.

### Cross-talk between γδ T cell and microbiota

The microbiota plays a crucial role in regulating T cell immunity.^662^ The dynamic interaction between commensal microbiota and T cells influences the maturation, differentiation, and effector function of T cells in various lymphoid tissues and organs.^663–665^ The microbiota provides essential signals and antigens for T cell activation and differentiation.^666–669^ Notably, specific bacterial species can induce the production of Tregs, contributing to immune tolerance and counteracting excessive inflammation.^670^ Microbiota-derived metabolites, such as SCFAs, play a role in promoting immune cell differentiation and function.^667,671–675^ Particularly during early life, the microbiota aids in the maturation of T cells and shapes their functional repertoire. Imbalances in gut microbiota composition, known as dysbiosis, are associated with alterations in T cell populations and functions, leading to immune dysregulation and increased susceptibility to diseases, including cancer, infections, and autoimmune disorders.^676,677^ Importantly, the interaction between the microbiota and T cells is reciprocal, with both components collaborating to establish a delicate balance critical for maintaining immune homeostasis. For instance, antibiotic (ABX) treatment or a low dietary fiber intake can induce alterations in the gut microbiota, which can contribute to cancer resistance to ICIs.^678–686^ The effectiveness of ICI immunotherapies is closely linked to the gut microbiome.^686–689^ Additionally, fecal microbiota transplantation (FMT) from responders has been shown to enhance the efficacy of anti-PD-1 therapy in cancer patients.^690,691^ Moreover, FMT from healthy donors could also be beneficial for patients with refractory ICI-induced colitis.^692^ Therefore, targeting the microbiota has emerged as a new and complementary treatment approach for cancer and autoimmune diseases.^693–695^ Recent studies have also indicated that the response and toxicity of CD19-CAR-T cell cancer immunotherapy are associated with the gut microbiome.^696^ Maintaining a non-antibiotic-disrupted gut microbiome is essential for the clinical efficacy of CD19-CAR-T cell cancer immunotherapy.^697^

Given the abundance of γδ T cells in peripheral tissues such as the skin, intestines, and lungs, which are also rich in commensal microbiota known to closely regulate γδ T cell functions,^470,473,698^ it is crucial to assess the impact of the commensal microbiota on the differentiation and effector functions of γδ T cells. This evaluation is essential for formulating effective therapeutic strategies.

### Paths for further improving γδ T cell therapy efficacy

#### Long-term transfer

A more important concern is how to increase the clinical efficacy of allogeneic Vδ1 or Vδ2 T cell-based cancer immunotherapy, as well as how to re-energize γδ T cells or maintain their long-term persistence. In our study, we discovered a drastic loss of Vδ2 T donor population 2 weeks after cell transfer, implying apoptotic cell death and exhaustion of donor cells. Since only those cancer patients who received multiple infusions had a higher probability to have better life quality and to survive longer, we thus propose that applying adoptive transfer regularly over extended time periods, at least until the time point of complete tumor remission or normalization of serum tumor makers, might be required. Moreover, we anticipate that allogeneic Vδ2 T cells will be an optimal clinical medicine for postoperative immune reconstitution of cancer patients, because of their dual properties of combining potent cytotoxicity with the ability to present antigens.

#### Engineering modifications of γδ T cells

Although our published research indicates the promising clinical efficacy of allogeneic γδ T cells derived from healthy donors,^11,12^ it is important to acknowledge the challenges posed by the exhaustion of tumor-infiltrating γδ T cells.^243^ This phenomenon serves as a reminder that even infused allogeneic γδ T cells could experience functional depletion upon infiltrating the complex tumor microenvironment. In light of this, engineering modifications of γδ T cells present a compelling avenue to surmount this hurdle. These modifications offer an innovative strategy to create off-the-shelf products endowed with enhanced anti-tumor activity and prolonged survival within the tumor microenvironment.

In the current landscape, multiple approaches to engineering γδ T cells have emerged, each holding considerable potential. For instance, CAR-γδ T cells,^25–27,699,700^ leveraging chimeric antigen receptors to confer γδ T cells with the ability to be more specifically target tumor-associated antigens, are one of frontiers of engineering γδ T cells. The representative applications of CAR-γδ T cells in clinical are briefly summarized in Table 2. On a different front, the creation of Gene-Modified Chemotherapy-Resistant γδ T cells^701–703^ equips these cells with the resilience to withstand the cytotoxic effects of chemotherapy agents, rendering them more effective agents for combination therapies, and the related clinical trial is posted either (NCT05664243). Another noteworthy advancement is the development of γδ T Cell Bispecific Antibody Adapters.^634,635,637,640,704,705^ These adapters bridge TCRs of γδ T cells and surface antigens tumor cells,^279^ facilitating direct and potent interactions between the two cell types in patients. Alternative paths to engineering γδ T cells like transferring γδ TCRs to αβ T cells,^706^ γδ TCR-T Cells (genetic modifications of the TCR),^707^ are also proposed and under investigation either. As for Antibody-Coupled γδ T Cells, which is based on the newly emerged Antibody-Coupled T Cell Receptor technique by utilizing the power of antibody-antigen interactions to enhance the targeting precision of γδ T cells toward tumor cells, are currently not documented yet.

The clinical significance of these engineered modifications is substantial. They offer the potential to overcome the limitations posed by exhaustion within the tumor microenvironment, amplifying the therapeutic impact of γδ T cells in cancer treatment. Looking ahead, the future applications of engineered γδ T cells extend beyond cancer. The lessons learned from these strategies could pave the way for novel therapies in infectious diseases, autoimmune disorders, and more. As research in this field progresses, engineered γδ T cells hold the promise of revolutionizing the landscape of immunotherapy, ushering in a new era of targeted and potent treatments.

#### Allogeneic γδ T plus existing therapeutic regimens

An additional strategy to further elevate the clinical efficacy of allogeneic γδ T cells is to combine them with other cancer treatment strategies such as chemotherapies and metformin^527,533,708,709^ which may help to relieve the TME pressures on donor γδ T cells and enhance their efficacy and persistence in the long-term. According to our previous work,^243^ TME-challenged γδ T cells express higher levels of lymphocyte activation gene 3 (LAG3) rather than other types of immune checkpoint molecules, and we thus propose that combination of allogeneic γδ T cell plus anti-LAG3 mAb will further greatly enhance the efficacy. Given the fact that PDL1 is routinely upregulated in tumor cells, the triple combo medicine γδ T cell, anti-LAG3 mAb, and anti-PD1 or anti-PDL1 mAb should be a better choice. Furthermore, in the context of TME stress, infused γδ T cells would gradually lose their chemotactic capability and thus could not migrate toward the tumor site. In this respect, various formats of bispecific antibodies are in development which will initiate a new direction for γδ T cell application.^704^ Additionally, combinations with other treatments including radiotherapy, interventional therapy, agonistic anti-BTN3A mAb, bispecific antibodies, or intratumoral application of zoledronate also greatly expand horizons of clinical applications of allogeneic γδ T cells.^335,633,636,710^

#### Allogeneic γδ T plus FMT

The gut microbiota, which plays a critical role in shaping the immune system and influencing diverse physiological processes such as tumorigenesis,^693,711–714^ has been demonstrated to orchestrate with immune responses of γδ T cells.^470^ The understanding of immune remodulation of gut microbiota emphasizes the potential of FMT as a complementary treatment alongside γδ T cells in tumor therapy. By transferring gut microbiota from a healthy donor to the recipient’s gastrointestinal tract, FMT takes advantage of the microbiota’s ability to impact tumor development and response to therapy.^679,693,712–714^ Currently, the exact mechanisms underlying FMT’s effects in tumor therapy are not fully understood but likely involve the interplay between the gut microbiota, immune cells, and the tumor microenvironment. The gut microbiota has been implicated in regulating immune cell activation, differentiation, and function, including γδ T cells.^470^ Therefore, the incorporation of FMT as an adjunctive treatment strategy could provide a promising avenue for improving the outcomes of allogeneic γδ T cell-based tumor immunotherapy.

### Detour the remaining technical roadblocks for γδ T cells

In the realm of functional research, the progress of functional research on γδ T cells has considerably lagged behind that of αβ T cells, primarily due to the absence of a specific gene conditional knockout mouse model for γδ T cells. This absence can be attributed to multiple factors, including the intricate and less understood nature of γδ T cell development within the thymus. Unlike αβ T cells, which have a well-defined developmental pathway and specific markers, γδ T cell development is characterized by its complexity and limited understandings. The process involves multiple subsets and distinct genetic programs influenced by TCR gene rearrangement, signaling networks, and interactions with thymic stromal cells, as discussed above.

The lack of a definitive marker or transcription factor^91,95,141,145^ exclusive to γδ T cells poses challenges in designing gene conditional knockout models specific to this subset. Furthermore, γδ T cells represent a smaller population within the thymus compared to αβ T cells, complicating the generation of targeted knockout models. Their lower abundance and absence of unique markers or genes pose difficulties in selectively targeting and manipulating their development using current conditional knockout strategies. Furthermore, the intricate interaction between γδ T cells and the thymic microenvironment adds another layer of complexity to the situation. Thymic stromal cells play a crucial role in supporting γδ T cell development and maturation through various signaling pathways and interactions. Disrupting a specific gene in thymic stromal cells may have unintended consequences on multiple T cell subsets, including αβ T cells, making it challenging to achieve selective knockout of γδ T cells.

Despite the challenges involved, ongoing efforts are being made to overcome the existing technical obstacles and establish targeted gene knockout mouse models for γδ T cells. The Kamiya group recently reported a novel approach for generating specific gene knockout mice in γδ T cells, introducing a detour paradigm.^90^ Their strategy involves the creation of mice with targeted gene deficiencies, followed by the isolation of γδ T cells from these mice and subsequent adoptive transfer into TCRγ-KO mice. This approach enables the development of specific gene knockout mouse models to study the function of a particular gene in γδ T cells. While this method has its limitations, it provides researchers with a valuable tool to explore the role of specific genes in the context of γδ T cell biology. Meanwhile, the ongoing progress in the development of humanized mouse models offers possibilities for in-depth investigating γδ T cells as well. This entails the utilization of appropriate murine models to investigate human γδ T cells, for instance the human TCR transgenic mice. Complementing this approach, it may be advantageous to incorporate transgenic expression of human BTN molecules. These combined efforts are poised to provide valuable insights into the complex biology of γδ T cells in a context closely mirroring human immunology.

## Closing remarks

The field of immunotherapy is continuously advancing, and among the emerging therapeutic strategies, allogeneic γδ T cells transfer have gained significant attention as a promising avenue for future immunotherapies. The unique properties of γδ T cells, such as their potent cytotoxicity, ability to recognize a broad range of antigens in MHC-independent manner, and potential for immunomodulation, make them attractive candidates for combating various diseases. To fully harness the therapeutic potential of γδ T cells, a deeper understanding of their underlying molecular mechanisms is essential. One area of research that warrants further exploration is thymus development, which plays a crucial role in shaping the repertoire and functional diversity of γδ T cells. Investigating the intricate processes involved in γδ T cell maturation and selection within the thymus will shed light on their ontogeny and help unravel the complex interplay between different subsets of γδ T cells.

Another aspect that requires closer examination is the plasticity of effector functions in γδ T cells, particularly in the context of disease microenvironment. γδ T cells possess the ability to exhibit diverse effector phenotypes, including cytotoxicity, cytokine production, and immunoregulatory functions. Understanding the factors that govern the plasticity of γδ T cell effector functions and the molecular cues that drive their differentiation into specific functional subsets will be crucial for optimizing their therapeutic applications.

In the realm of clinical trials, the integration of γδ T cell-based immunotherapy as adjuvant applications holds great promise. Combining γδ T cell therapy with existing treatment regimens, such as chemotherapy or checkpoint blockade, has the potential to synergistically enhance anti-tumor responses and improve patient outcomes. Additionally, exploring innovative strategies like FMT, which can modulate the gut microbiome and influence γδ T cell functionality, may further enhance the therapeutic efficacy of γδ T cell-based immunotherapies.

In conclusion, the evolving landscape of immunotherapy highlights allogeneic γδ T cell transfer as a promising avenue for future treatments. Leveraging γδ T cells’ unique attributes, such as their versatile antigen recognition and immunomodulatory potential, presents exciting therapeutic possibilities. To unlock their full potential, a deeper comprehension of γδ T cell development and plasticity is imperative. Investigating thymus-driven maturation and understanding effector function plasticity within disease contexts will guide their optimal use. Integrating γδ T cell therapy into clinical approaches, including synergistic combinations with existing treatments and innovative strategies like microbiome modulation, holds great potential. This ongoing scientific exploration promises personalized and effective immunotherapies. By unraveling the intricacies of γδ T cell biology, interactions with microenvironments, and their therapeutic applications, we are poised to revolutionize precision medicine and fully harness γδ T cells’ therapeutic prowess.
